# MiR-483-5p downregulation alleviates ox-LDL induced endothelial cell injury in atherosclerosis

**DOI:** 10.1186/s12872-023-03496-1

**Published:** 2023-10-27

**Authors:** Hezhong Zhu, Hui Liang, Zhen Gao, Xiaoqiao Zhang, Qian He, Chaoyong He, Chao Cai, Jiajuan Chen

**Affiliations:** 1 Department of Geriatrics, Taihe Hospital, Hubei University of Medicine, Shiyan, 442000 China; 2 Department of Cardiology, Taihe Hospital, Hubei University of Medicine, No. 32 Renminnan Road, Shiyan, 442000 China

**Keywords:** Atherosclerosis, Endothelial injury, MiR-483-5p, Autophagy, TIMP2

## Abstract

**Background:**

In light of the abnormal expression of microRNA (miR-483-5p) in patients with atherosclerosis (AS), its role in vascular endothelial cell injury was explored. And the mechanisms related to autophagy were also elucidated.

**Methods:**

Human umbilical vein endothelial cells (HUVECs) were given 100 mg/L ox-LDL to induce endothelial injury. Cell transfection was done to regulate miR-483-5p levels. Cell viability and apoptosis were detected. qRT-PCR was employed for the mRNA levels’ detection.

**Results:**

Autophagic flux impairment of HUVECs was detected after ox-LDL treatment, along with the upregulation of miR-483-5p. Ox-LDL inhibited cell viability and promoted cell apoptosis, but these influences were changed by miR-483-5p downregulation. MiR-483-5p downregulation decreased the mRNA levels of IL-1β, IL-6, ICAM-1 and VCAM-1. 3-MA, the autophagy inhibitor, reversed the beneficial role of miR-483-5p downregulation in ox-LDL-induced HUVECs’ injury. TIMP2 acts as a target gene of miR-483-5p, and was downregulated in HUVEC models.

**Conclusion:**

MiR-483-5p downregulation alleviated ox-LDL-induced endothelial injury via activating autophagy, this might be related to TIMP2.

**Supplementary Information:**

The online version contains supplementary material available at 10.1186/s12872-023-03496-1.

## Background

Atherosclerosis (AS) is a chronic inflammatory disease that is characterized by the accumulation of lipids, cholesterol, calcium, and cellular debris on the inner lining of blood vessels [1]. AS is the common precipitating factor of cardiovascular and cerebrovascular diseases such as ischemic heart disease, stroke and peripheral vascular disease, and even death [2]. AS is caused by many factors, among which vascular endothelial cell (VEC) injury is the initial link to the occurrence and development of AS [3]. Autophagy is a key process for cells to meet metabolic requirements during growth, differentiation and development [4]. It mainly transports damaged organelles and denatured proteins to lysosomes for digestion and degradation [5]. The data showed that in the process of widespread VEC autophagy, insufficient or excessive autophagy could induce endothelial cell apoptosis, thus initiating and accelerating the formation of AS [6]. In the physiological state, VEC promotes lysosomal degradation of oxidized low-density lipoprotein cholesterol (ox-LDL) by activating autophagy to resist its induced injury [7]. The exceedance of ox-LDL accumulation will lead to VEC damage further contributing to the development of AS [8]. At this time, VEC autophagy is beneficial for the inhibition of AS [9]. Therefore, when ox-LDL accumulation exceeds the metabolic capacity of cells, VEC damage induces AS, and at this time, the occurrence of AS can be reduced by increasing VEC autophagy.

MicroRNAs (miRNAs) are a class of small non-coding single-stranded RNAs. It specifically recognizes the 3’-untranslated region (3’-UTR) of target gene mRNAs and mediates the target genes’ degradation or translation inhibition, thus down-regulates their expression [10]. Functionally, miRNAs participate in a variety of biological processes such as cell proliferation, differentiation, apoptosis, autophagy, and even the pathophysiology of AS [11, 12]. Recent studies have demonstrated that some miRNAs have obvious regulatory effects on VEC autophagy [13, 14]. For example, Pankratz et al. have reported the beneficial effect of miR-100 on endothelial injury, which is related to its promotive role in endothelial autophagy and following anti-inflammatory function [15]. MiR-483-5p is an evolution-highly conserved miRNA, it has been widely reported to be correlated with the onset of human diseases [16]. Recently, miR-483-5p is reported to be involved in the regulation of AS development [16]. Clinically, high levels of miR-483-5p are identified in the serum of AS cases [17]. However, its underlying mechanism has not been elucidated.

In the occurrence and development of AS, the damage and dysfunction of VECs are the core and initial steps, and the change of autophagy level of VECs can regulate various functions of VECs. Therefore, searching for regulatory factors of autophagy in VECs has become a new idea for the early clinical treatment of AS. However, the regulatory mechanism of autophagy in VECs has not been elucidated. Notably, a close correlation of miR-483-5p with autophagy has been proposed, which is related to cisplatin-induced acute kidney injury. Therefore, in the current study, human umbilical vein endothelial cells (HUVECs) were recruited for the in vitro experiments. The role of miR- miR-483-5p in the cell dysfunction of HUVECs induced by ox-LDL was explored. Furthermore, the involvement of autophagy in the underlying mechanism was examined. It was assumed that miR-483-5p might regulate ox-LDL-induced endothelial injury via mediating autophagy.

## Methods

### Cell culture and model establishment

Human umbilical vein endothelial cells (HUVECs) were purchased from ScienCell Research Laboratories (Carlsbad, CA, USA). HUVECs were incubated with RPMI 1640 medium (Solarbio, Beijing, China) containing 10% FBS in an incubator containing 5% CO_2_ at 37 °C. When the cell growth reached 90% confluence, the medium was discarded, then PBS and trypsin were added respectively for cell washing and digestion, and sub-culturation was performed. To mimic the AS condition in vitro, HUVECs were given 100 mg/L ox-LDL (Yiyuan Biotech, Guangzhou, China) and cultured for 24 h.

### Cell transfection

To regulate the levels of miR-483-5p in HUVECs, miR-483-5p mimic (50nM, 5’-AAGACGGGAGGAAAGAAGGGAG-3’) or inhibitor (50 nM, 5’-CTCCCTTCTTTCCTCCCGTCTT-3’) was transfected into HUVECs. And cells transfected with its negative control sequence (miR-NC) were applied as the control group. The small-interference RNA of TIMP2 (si-TIMP2; 5’-TTGAGATTATCATAAGCAGAG-3’) was used for the gene knockdown assay, and the negative control sequence (si-NC; 5’-ACGAGACACGAACGGAGAATT-3’) was used for the reference. All sequences were chemically synthesized by Shanghai GenePharma Co (Shanghai, China). The cell transfection was performed using Lipofectamine 2000 (Invitrogen, Carlsbad, CA, USA) [18]. After transfection in 37℃ cell incubator for 4-6 h, the fresh medium was replaced.

### CCK-8 assay

CCK-8 kit (Bestbio, Shanghai, China) was employed for the cell viability test [19]. HUVECs were inoculated in 96-well culture plates and added with 10 μl CCK-8 at 0,24, 48 and 72 h, respectively. After continued culture for 4 h, the absorbance values at 450 nm of each group of cells were detected by enzyme marker. According to the obtained OD values, the variation curves of OD values at different time points were drawn.

### Flow cytometry assay

Annexin V-FITC Apoptosis Detection Kit (Yeasen, China) was employed for the measurement of HUVECs’ apoptosis [20]. The cells of each group were collected and re-suspended in the binding buffer. 5 μl Annexin V and 10 μl propidium (PI) reagents were added, and the cells were incubated against light for 15 min. The apoptosis rate was detected by flow cytometry within 30 min.

### Western blot

The protein levels were detected by western blotting [21]. Cells of each group were collected and 250 μl protein lysate was added for protein extraction. The BCA Protein Quantitative Detection kit (Servicebio, Wuhan, China) was applied for the protein quantitative detection. The 12% SDS separation gel was used for electrophoresis in constant pressure mode of 70 ~ 120 V. After the membrane was transferred, the primary antibody of each protein (LC3II, P62) was added, then the secondary antibody was added after overnight culture at 4 ℃. The protein bands were exposed in the UVP gel imaging system (UVP, MA, USA), and the Image-J software was used to scan the gray values of each band. The relative protein expression was calculated according to the gray values.

### qRT-PCR

Total RNA was extracted by Trizol method, and RNA concentration and A260/A280 were detected by ultra-micro spectrophotometer (Implen, Munich, Germany). After confirming that the RNA of each sample was qualified, reverse transcription was performed according to the operation procedure of the reverse transcription kit, and the obtained cDNA was used for qPCR. U6 or GAPDH were used as internal reference for the miR-483-5p or genes (IL-1β, IL-6, ICAM-1, VCAM-1) respectively. And the relative mRNA levels were calculated by 2^−ΔΔCt^ method [22]. The PCR reaction conditions were 95℃ predenaturation for 30 s, 95℃ denaturation for 5 s, 60℃ for 34 s, and a total of 40 cycles were done. The primers were designed based on the previous references [23–25] and synthesized by Shanghai Bioasia Biotech (Shanghai, China), and the sequences were listed in Table 1.

**Table 1:** The primer sequences used in the present study

Primers	Sequences (5’-3’)	
miR-483-5p	Forward	GCCGAGAAGACGGGAGGAAA
	Reverse	CTCAACTGGTGTCGTGGA
IL-1β	Forward	CACAGCAGCACATCAACAAG
	Reverse	GTGCTCATGTCCTCATCCTG
IL-6	Forward	CCTGCGTTTAAATAACATCAGCTTTAGCTT
	Reverse	GCACAATGTGACGTCGTTTAGCATCGAA
ICAM-1	Forward	GGAACCCATTGCCCGAGC
	Reverse	GGTGAGGATTGCATTAGGTC
VCAM-1	Forward	CTGCTCAAGTGATGGGATACCA
	Reverse	AGGCTGCAGTTCC-CCATTATT
U6	Forward	CTCGCTTCGGCAGCACA
	Reverse	AACGCTTCACGAATTTGCGT
GAPDH	Forward	TCCACTGGCGTCTTCACC
	Reverse	GGCAGAGATGATGACCCTTTT

### Luciferase reporter assay

The target sequence between miR-483-5p and TIMP2 was predicted by TargetScan 7.0. Wild-type or mutant (WT or MUT) luciferase reporter vectors of 3’ -UTR-TIMP2 containing miR-483-5p binding site sequences or mutant sequences were provided by Shanghai Gima Pharmaceutical Technology Co., LTD. WT/MUT-TIMP2 was co-transfected with miR-483-5p mimics or miR-483-5 inhibitors into HUVECs, respectively. After 48 h of culture, cells were collected and the luciferase activity of cells in each group was detected [26].

### Statistical analysis

SPSS 20.0 was used for data processing, and GraphPad Prism 7.0 was employed for the figure drawing. All experiments were repeated three times. Differences between the means of the two groups were assessed using Student’s t-test. Tests of differences among multiple (more than two) groups were performed via one-way analysis of variance (ANOVA) analysis. *P* < 0.05 was considered statistically significant.

## Results

### Ox-LDL-induced autophagic flux impairment of HUVECs accompanied by the upregulation of miR-483-5p

**Fig. 1:**
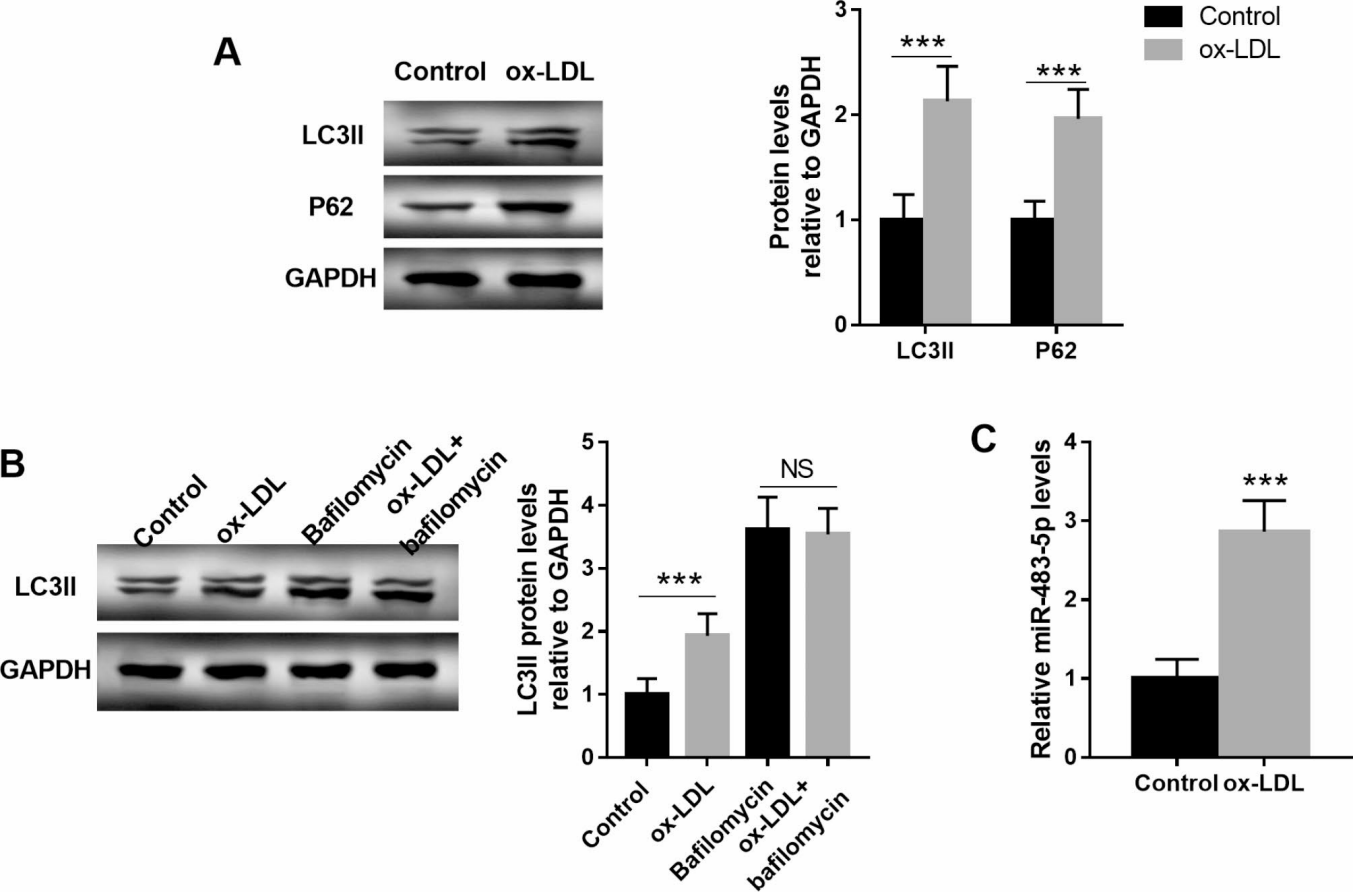
Ox-LDL (100 mg/L) induced autophagic flux impairment of HUVECs accompanied by the upregulation of miR-483-5p. **A**. Elevated expression of LC3II and P62 was detected in ox-LDL-treated HUVECs. The full-length blots are presented in Supplementary Fig. 1A and the blots were cut prior to hybridisation with antibodies during blotting. **B**. LC3II protein levels showed no significant difference between Baf A1 group and ox-LDL + Baf A1 group, but it was significantly elevated in ox-LDL group compared with the control group. The full-length blots are presented in Supplementary Fig. 1B. The blots were cut prior to hybridisation with antibodies during blotting. **C**. MiR-483-5p was elevated in ox-LDL group compared with the control group. *** means *P* < 0.001

The autophagy of HUVECs was detected after ox-LDL treatment. As observed from Fig. 1A, elevated expression of LC3II and P62 was tested in ox-LDL-treated HUVECs (*P* < 0.001). Bafilomycin A1 (Baf A1) is a late-stage inhibitor of autophagy. It is well known that LC3B-I/II transformation of cells after the use of late-stage autophagy inhibitors is currently the gold index of autophagy flux detection. The western blot results showed that no significant difference was detected for LC3II protein levels between Baf A1 group and ox-LDL + Baf A1 group, illustrating that ox-LDL did not affect the synthesis of autophagosomes (Fig. 1B, P > 0.05). But LC3II protein levels were significantly elevated in ox-LDL group compared with the control group, demonstrating that ox-LDL inhibited the degradation of autophagosomes (Fig. 1B, P < 0.001). All findings proposed that ox-LDL induced autophagic flux impairment of HUVECs. Moreover, miR-483-5p levels were also compared between control group ox-LDL groups, and a significant difference was detected (Fig. 1C, P < 0.001).

### MiR-483-5p downregulation reversed ox-LDL-induced endothelial injury

**Fig. 2:**
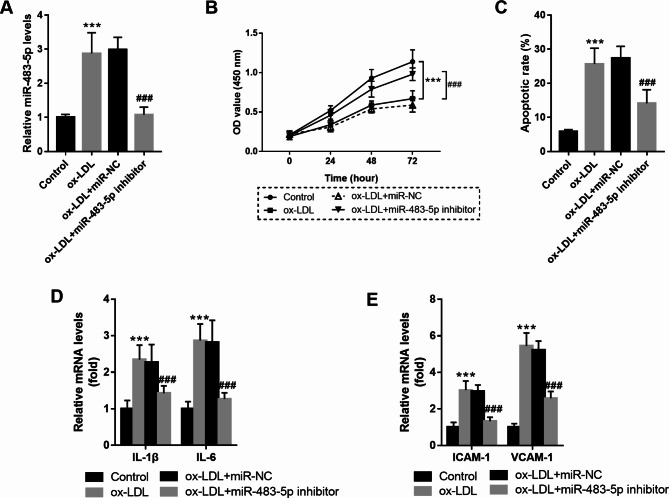
MiR-483-5p downregulation reversed ox-LDL (100 mg/L) induced endothelial injury. **A**. ox-LDL treatment contributed to the increase of miR-483-5p, but this trend was reversed after miR-483-5p inhibitor transfected into cells. **B**. MiR-483-5p downregulation promoted HUVECs’ viability. **C**. MiR-483-5p downregulation suppressed HUVECs’ apoptosis. **D**. ox-LDL elevated the mRNA levels of IL-1β, IL-6, which were abolished by miR-483-5p inhibitor. **E**. ox-LDL elevated the mRNA levels of ICAM-1 and VCAM-1, which were abolished by miR-483-5p inhibitor. *** means *P* < 0.001 compared with the control group; ### means *P* < 0.001 compared with ox-LDL group

To discover the function of miR-483-5p in HUVECs’ function, miR-483-5p levels were regulated via cell transfection. As seen in Fig. 2A, ox-LDL addition contributed to the increase of miR-483-5p, but this trend was reversed after the miR-483-5p inhibitor transfected into cells (*P* < 0.001). The CCK-8 and flow cytometry assay revealed that ox-LDL inhibited cell viability and promoted cell apoptosis, but these influences were changed by miR-483-5p downregulation (*P* < 0.001, Fig. 2B-C). In addition, in HUVECs, ox-LDL also elevated the mRNA levels of IL-1β, and IL-6, which were abolished by miR-483-5p inhibitor (*P* < 0.001, Fig. 2D). Similar changes were also detected for ICAM-1 and VCAM-1 mRNA (*P* < 0.001, Fig. 2E).

### MiR-483-5p downregulation remitted ox-LDL triggered HUVECs’ injury via stimulating autophagy

**Fig. 3:**
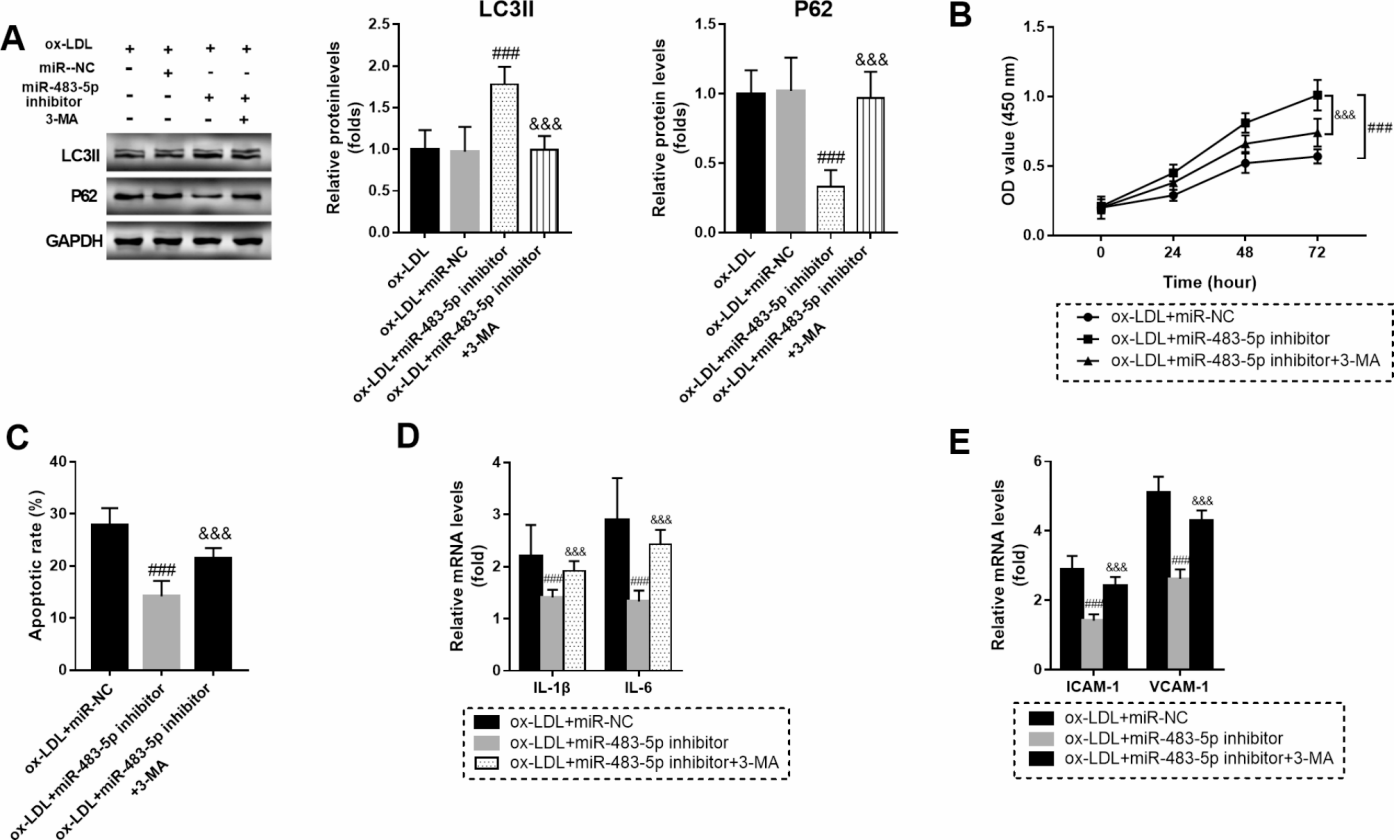
MiR-483-5p downregulation remitted ox-LDL (100 mg/L) induced HUVECs’ injury via motivating autophagy. **A**. miR-483-5p inhibitor significantly promoted the expression of LC3II but inhibited the expression of P62, but these effects were reversed by 3-MA. The full-length blots are presented in Supplementary Fig. 3A and the blots were cut prior to hybridisation with antibodies during blotting. **B**. 3-MA reversed the promoting effect of miR-483-5p downregulation on cell viability. **C**. 3-MA counteracted the effect of miR-483-5p downregulation on cell apoptosis. **D**. The suppressed mRNA levels of IL-1β, IL-6 were upregulated again by 3-MA. **E**. The suppressed mRNA levels of ICAM-1 and VCAM-1 were upregulated again by 3-MA. *** means *P* < 0.001 compared with the ox-LDL + miR-NC group; ^&&&^ means *P* < 0.001 compared with the ox-LDL + miR-483-5p inhibitor group

3-methyladenine (3-MA) is a classic autophagy inhibitor, which can inhibit the formation and development of autophagosomes. To discover the function of miR-483-5p in autophagy. HUVECs were cultured with 3-MA after miR-483-5p inhibitor transfection. As shown in Fig. 3A, miR-483-5p inhibitor significantly promoted the expression of LC3II but inhibited the expression of P62, but these effects were reversed by 3-MA (*P* < 0.001). Similarly, the promoting effects of miR-483-5p inhibitor on HUVEC cell viability and inhibiting effect on cell apoptosis were offset by 3-MA (*P* < 0.001, Fig. 3B-C). Besides, 3-MA also increased the mRNA levels of IL-1β, IL-6, ICAM-1 and VCAM-1, which has been suppressed by miR-483-5p inhibitor (*P* < 0.001, Fig. 3D-E).

### Targeting relation between TIMP2 and miR-483-5p

**Fig. 4:**
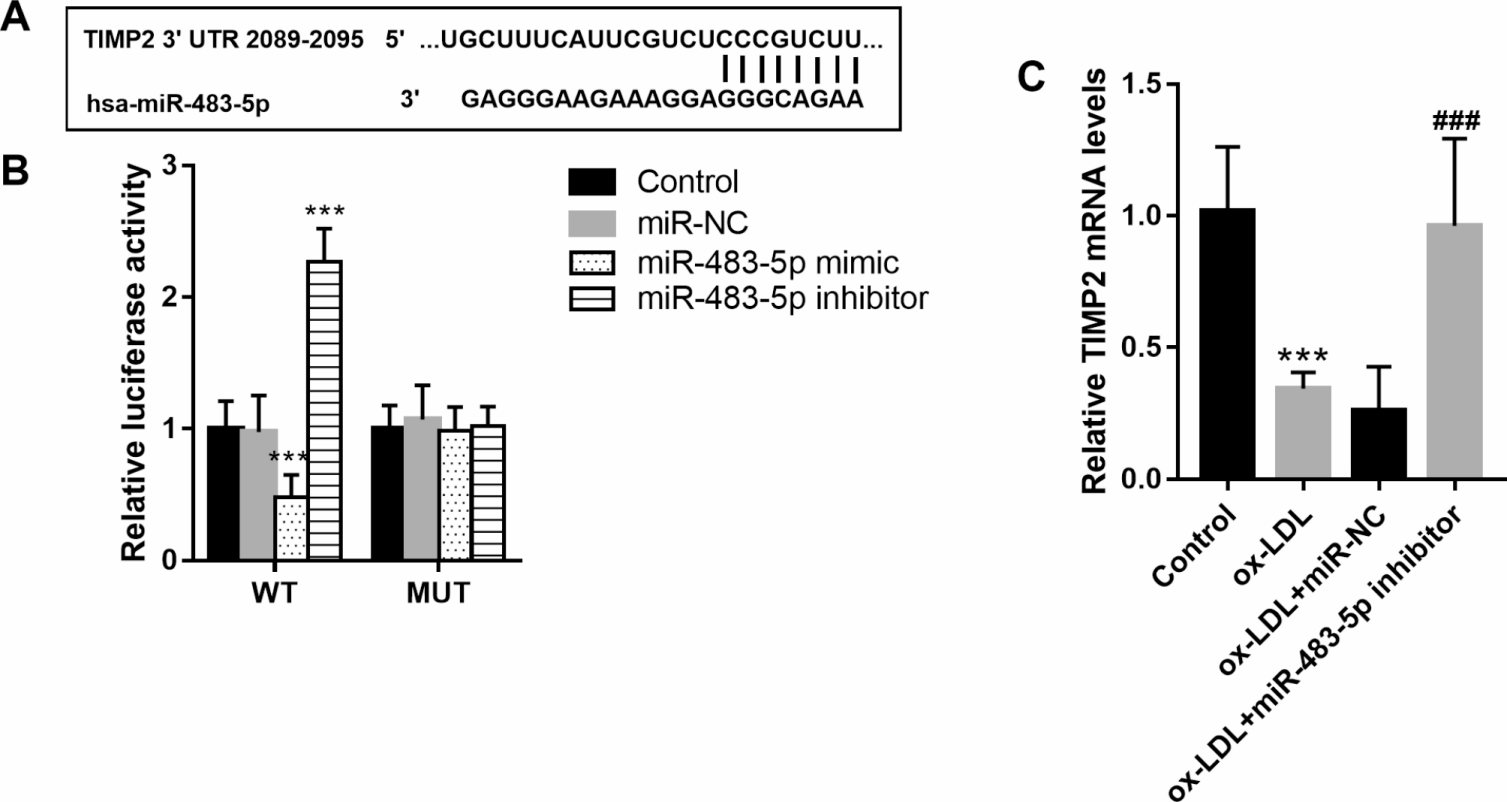
Target relation between TIMP2 and miR-483-5p. **A**. The target bindings sites between miR-483-5p and the 3’UTR of TIMP2 gene. **B**. MiR-483-5p overexpression inhibited the luciferase activity of HUVECs transfected with wild-type sequence of TIMP2, which was promoted by miR-483-5p downregulation. **C**. Decreased TIMP2 mRNA was detected in ox-LDL (100 mg/L) treated HUVECs. *** means *P* < 0.001 compared with the control group; ### means *P* < 0.001 compared with ox-LDL group

The target gene of miR-483-5p was preliminary verified in HUVECs. Figure 4 A showed the target binding sites between miR-483-5p and the 3’UTR of the TIMP2 gene predicted by TargetScan 7.0. Besides, the luciferase activity assay demonstrated that miR-483-5p overexpression inhibited the luciferase activity of HUVECs transfected with a wild-type sequence of TIMP2, which was promoted by miR-483-5p downregulation (Fig. 4B). But miR-483-5p levels did not influence the luciferase activity of HUVECs transfected with the mutant sequence of TIMP2 (*P* > 0.05). In ox-LDL-treated HUVECs, decreased TIMP2 mRNA was detected (*P* < 0.001, Fig. 4C), which was enhanced by miR-483-5P downregulation.

## Discussion

Recently, miR-483-5p is reported to be involved in the regulation of AS development [16]. However, its underlying mechanism has not been elucidated. In the current study, HUVECs were treated with ox-LDL to establish AS cell models, the cell autophagy and miR-483-5p changes were detected. It was found that autophagic flux impairment of HUVECs occurred along with the upregulation of miR-483-5p. The rescue experiments illustrated that miR-483-5p downregulation can prevent endothelial dysfunction, and the mechanism is related to cell autophagy and the TIMP2 gene.

Autophagy is a highly conserved biological process that is widely present in various plants and animals and is essential for cellular stress responses [27]. In recent years, the role of autophagy in various cardiac and vascular diseases has been studied and reported [28]. In VECs, autophagy appears as a major protective mechanism [29]. As reported, ox-LDL is highly cytotoxic and damages endothelial cells. After endothelial cell injury, its brittleness and adhesion increase, which makes more low-density lipoproteins enter the subintimal deposition and oxidized into ox-LDL, forming a vicious circle [30]. In this study we explored, HUVECs induced culture with ox-LDL to mimic the endothelial cell injury, it can be seen that ox-LDL induced autophagic flux impairment of HUVECs. Interestingly, autophagic flux impairment of HUVECs occurred along with the upregulation of miR-483-5p. The findings prompted us to deduce that ox-LDL-induced autophagic flux impairment of HUVECs might be related to the dysregulation of miR-483-5p.

MiRNA is a newly discovered gene regulator in recent years [31]. MiRNA is implicated in the mediation of cell proliferation, differentiation, apoptosis and other biological processes by specifically downregulating the post-transcriptional expression of target genes [32]. As previously reported, multiple miRNAs have been identified to be at abnormal expression in clinical AS patients or AS cell or animal models [33]. In AS cases, miR-483-5p is pointed out to be at high expression compared with the controls [17]. The present study recruited HUVECs and explored the role of miR-483-5p in VEC dysfunction. It was found that ox-LDL treatment led to the upregulation of miR-483-5p in HUVECs, which was consistent with the clinical findings previously [17]. Through cell transfection, miR-483-5p levels were downregulated via miR-483-5p inhibitor. The cell experiments proved that ox-LDL suppressed HUVECs viability while stimulating cell apoptosis, but these influences were reversed by miR-483-5p downregulation. The injury of vascular endothelial cells by ox-LDL can activate the immune response and lead to the secretion of inflammatory factors, such as IL-6 and IL-1β [34]. Vascular endothelial dysfunction triggers the increase of cell adhesion factors, leading to the formation of local thrombus. In addition, the adhesion of monocytes prompts the release of chemokines, which further differentiate into macrophages and further activate the inflammatory response [35]. IL-6, IL-1β, ICAM-1 and VCAM-1 are the main pro-inflammatory cytokine and adhesion molecules in vascular endothelial cells, so their levels in HUVECs were detected. Consistent with the previous evidence, large releases of inflammatory cytokines and adhesion molecules were detected in ox-LDL-treated HUVECs. And miR-483-5p downregulation inhibited the release of related factors induced by ox-LDL in HUVECs. The findings supported the close relationship of miR-483-5p with endothelial dysfunction. Consistently, in pulmonary microvascular endothelial cells (PMVECs) cells, miR-483-5p is also determined to exacerbate LPS-induced cell inflammation and apoptosis [36], which supported our present findings in HUVECs.

In recent years, in vitro studies have reported the regulatory mechanism of miRNA on the autophagy of VECs, which provides a new idea for the regulatory mechanism of endothelial cell function in AS [37, 38]. A recent study about acute kidney injury (AKI) has reported the regulatory effect of miR-483-5p on tubular cell autophagy, which explains the possible mechanism of miR-483-5p in acute kidney injury [39]. In the present ox-LDL treated HUVEC models, it was found that the autophagic flux impairment of HUVECs occurred along with the upregulation of miR-483-5p, proposing the potential relationship between miR-483-5p and autophagy, as well as endothelial dysfunction. 3-methyladenine (3-MA) is a classical autophagy inhibitor, which mainly plays an inhibitory role in the formation and development of autophagosomes [40]. In the current study, the addition of 3-MA was applied to inhibit the HUVECs autophagy, in order to investigate the role of miR-483-5p in cell autophagy. The results demonstrated that 3-MA addition abolished the protective role miR-483-5p downregulation against endothelial dysfunction. The conclusion was made that miR-483-5p downregulation protected against ox-LDL-induced HUVECs damage via activating autophagy.

Previous studies have demonstrated that tissue inhibitor of metalloproteases 2 (TIMP2) is a candidate target of miR-483-5p through bioinformatics prediction and dual luciferase reporter gene analysis [41, 42]. Consistently, the target relation between miR-483-5p and TIMP2 was also confirmed in HUVECs via dual luciferase reporter gene analysis. TIMP2 is a member of TIMPs family, it is highest expressed in the mouse heart [43, 44]. It has been reported that the knockdown of TIMP2 is related to the progression of cardiovascular disease [45]. As Di Gregoli et al. reported, TIMP2 deficiency resulted in increased atherosclerotic plaque instability in ApoE -/- mice on a high-fat and high-cholesterol diet, and the mechanism was associated with increased MMP activity, increased degradation of matrix proteins, and increased macrophage accumulation and apoptosis [45]. Up-regulation of TIMP2 can inhibit the invasion of mononuclear macrophages into atherosclerotic lesions [45]. In the current study, downregulated mRNA levels of TIMP2 were observed in ox-LDL-treated HUVECs, which were negatively correlated with miR-483-5p levels. Furthermore, the role of TIMP2 in autophagy has also been determined. For example, in Mycobacterium tuberculosis (MTB) infected alveolar epithelial cells, overexpression of TIMP2 can promote the cell autophagy thereby suppressing inflammatory response [46]. Moreover, TIMP2 also plays a prominent role in the inflammatory response and adhesion of a variety of cells, including endothelial cells [47–49]. Endothelial cell dysfunction increases the expression of inflammatory factors such as cell adhesion factors and cytokines, which causes the adhesion and infiltration of inflammatory cells into the vascular wall and produces vascular inflammatory response. These pathological changes play an important role in the occurrence of AS [49]. Therefore, in light of the previous evidence and the present findings, we deduced that miR-483-5p downregulation might recover the endothelial dysfunction by promoting autophagic flux via targeting TIMP2. However, in this study, the role and mechanism of miR-483-5p were only preliminarily explored in vitro. Further in vitro experiments are necessary to verify its function. And the downstream mechanism needs to be further explored.

## Conclusions

In conclusion, autophagic flux impairment of HUVECs occurred after ox-LDL treatment along with the upregulation of miR-483-5p. MiR-483-5p downregulation remitted ox-LDL-induced endothelial injury via motivating autophagy. This finding may further deepen our understanding of the pathogenesis of AS, and may afford a possible therapeutic target for more effective clinical cure of AS However, the participation mechanism of TIMP2 needs to be further verified.

### Electronic supplementary material

Below is the link to the electronic supplementary material.

Supplementary Material 1

## Data Availability

The datasets used and/or analysed during the current study are available from the corresponding author on reasonable request.
