# Supplementary material for: MiR-483-5p downregulation alleviates ox-LDL induced endothelial cell injury in atherosclerosis
Source: BMC Cardiovasc Disord. 2023 Oct 27;23:521. doi: 10.1186/s12872-023-03496-1 (PMC10612234; doi:10.1186/s12872-023-03496-1)

**Figure 1A**

LC3II

Repeat 1 Repeat 2 Repeat 3


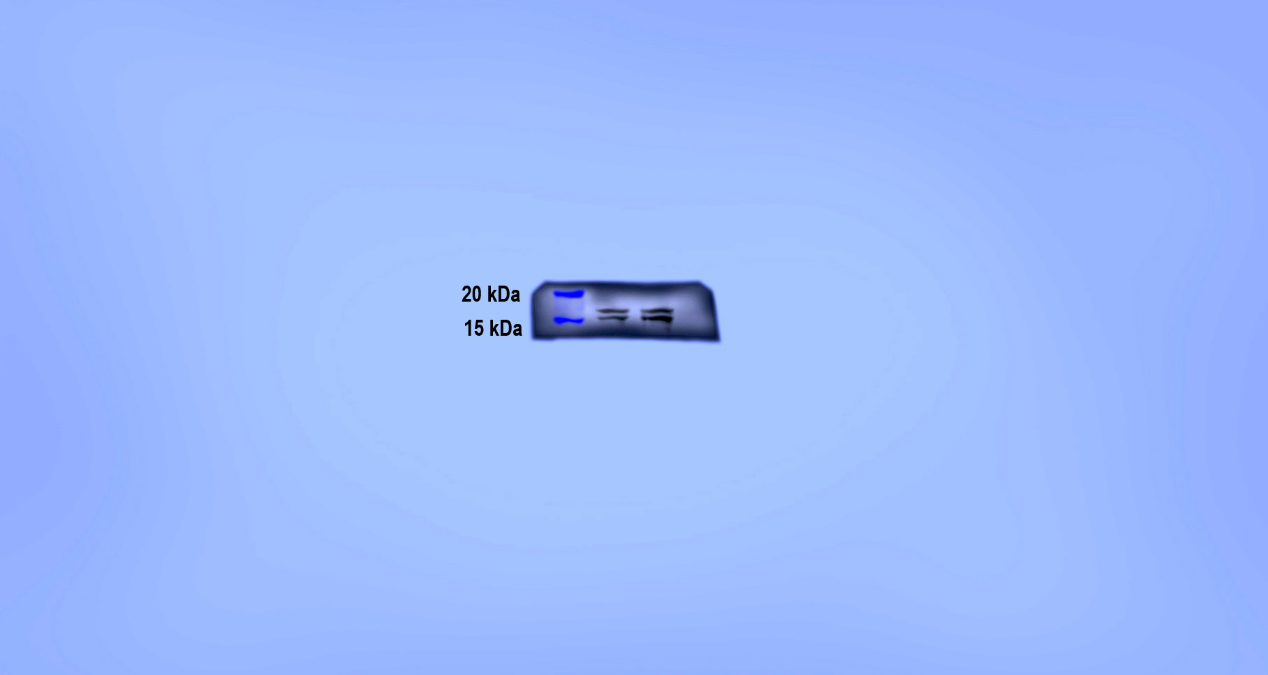

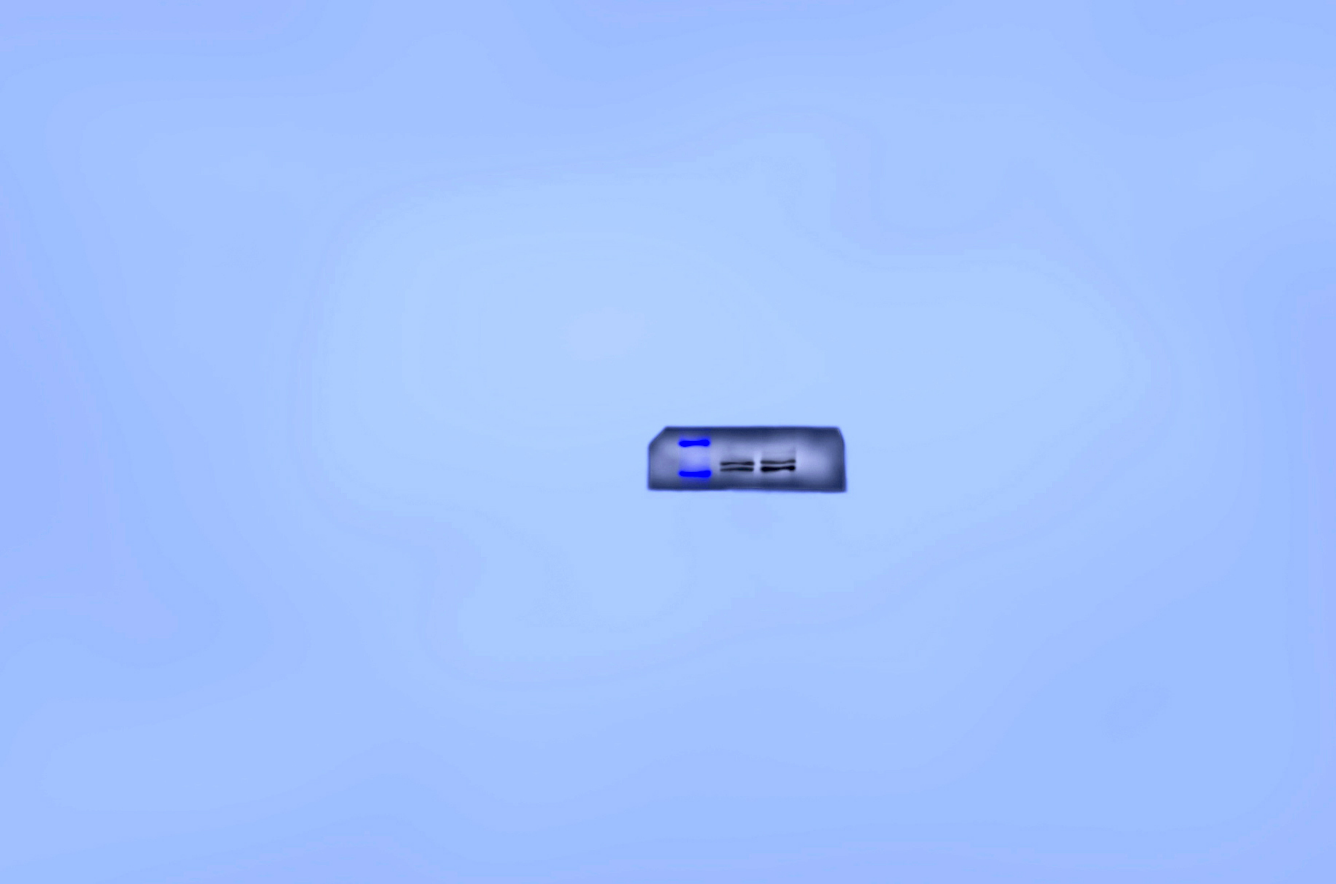

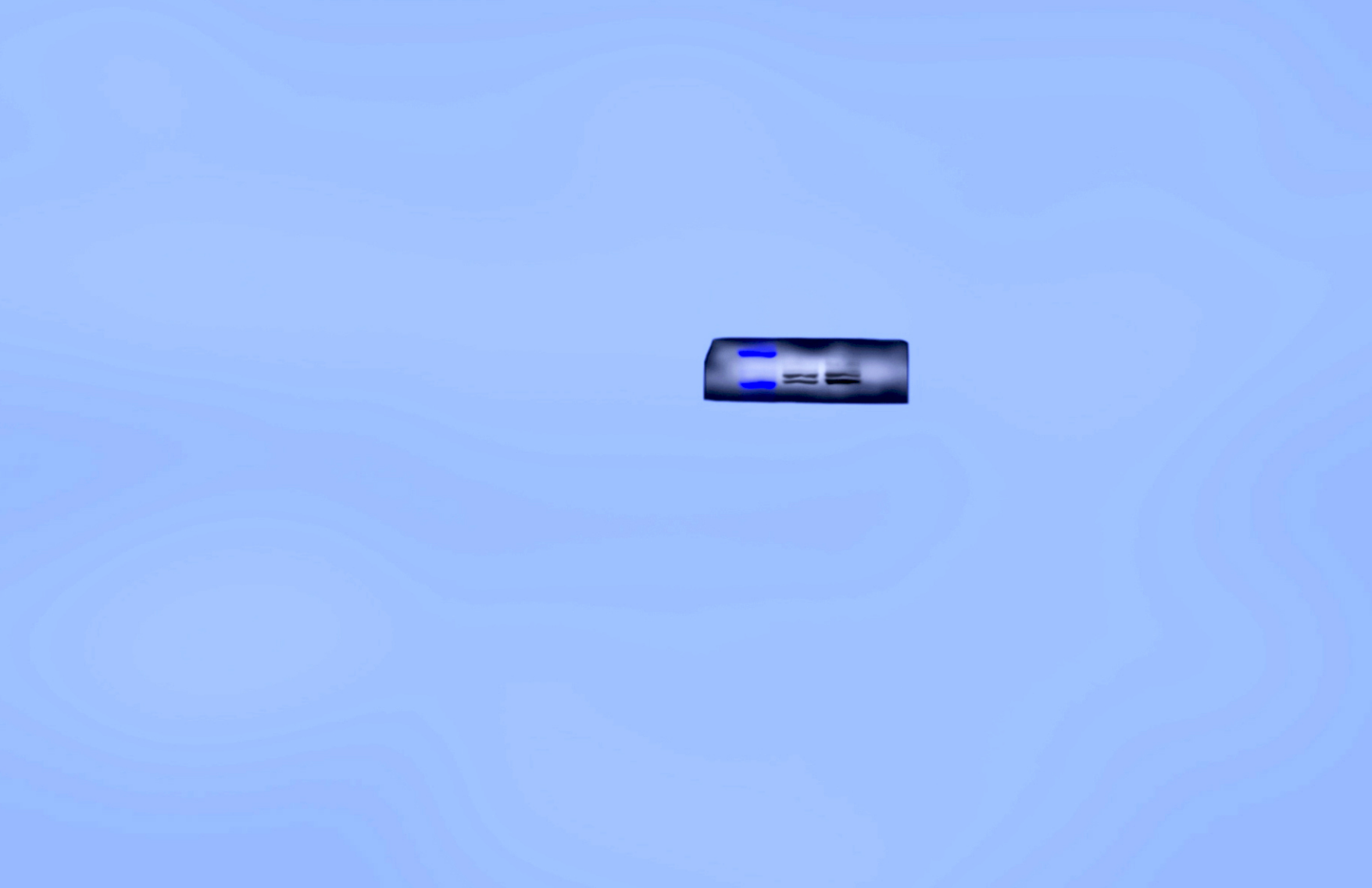


P62

Repeat 1 Repeat 2 Repeat 3


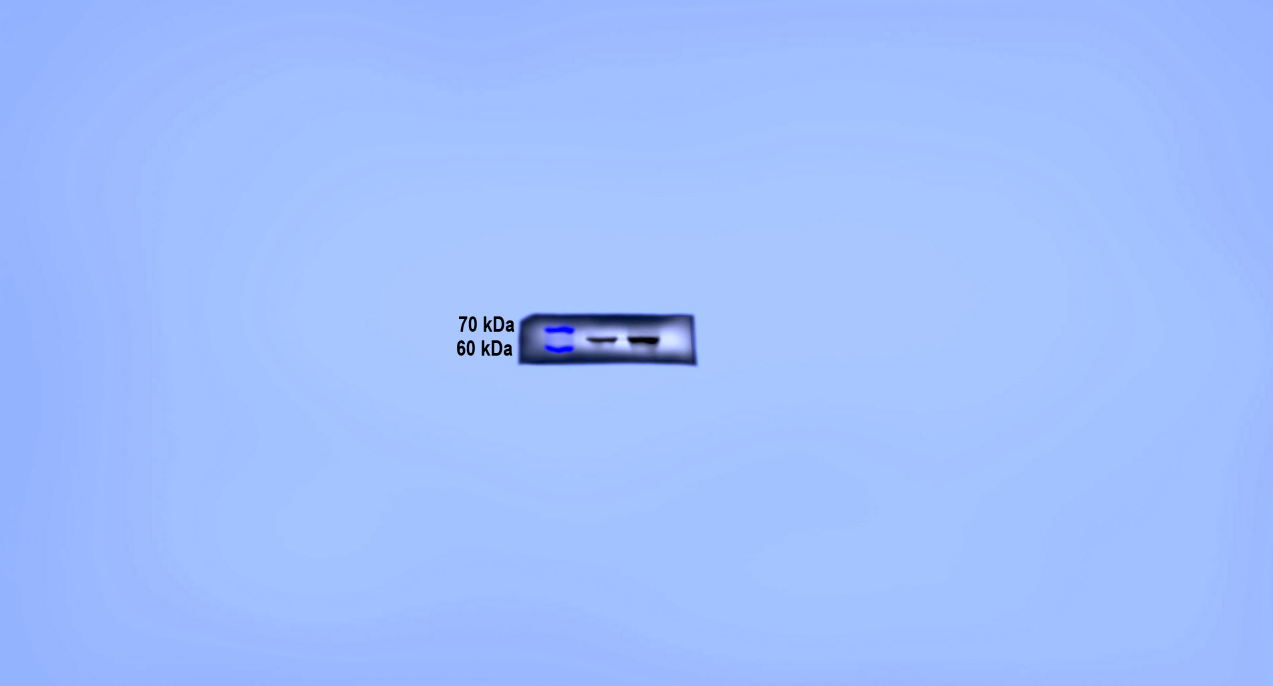

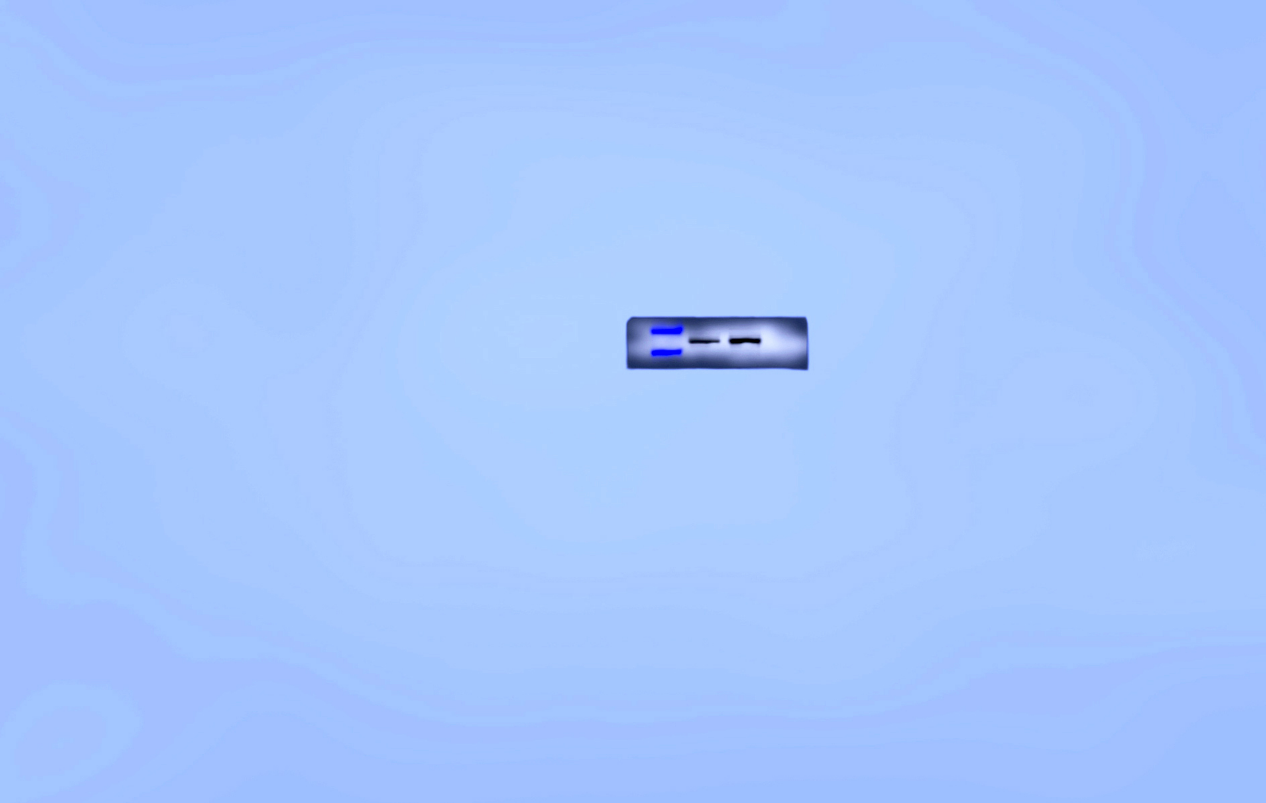

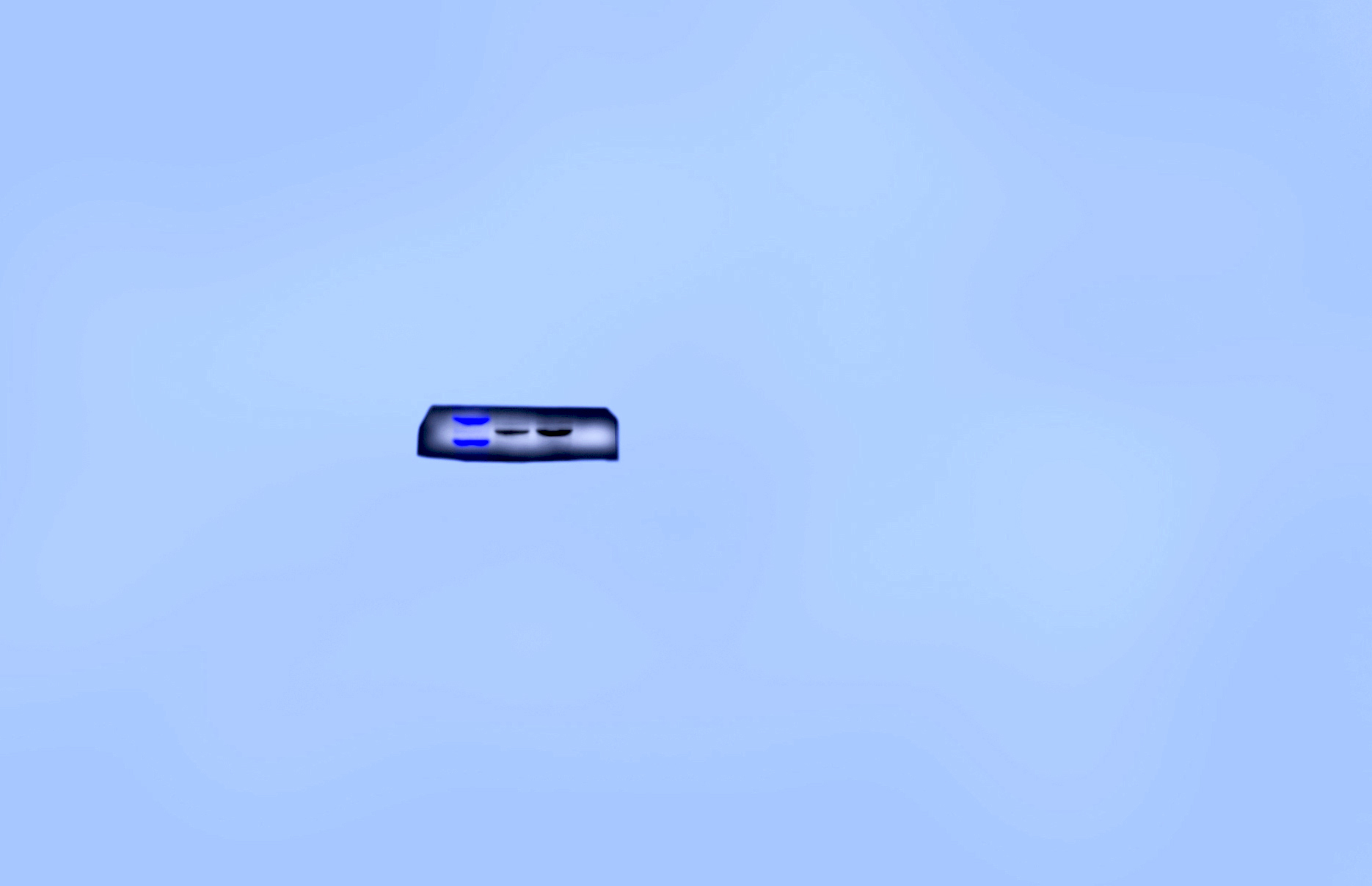


GAPDH

Repeat 1 Repeat 2 Repeat 3


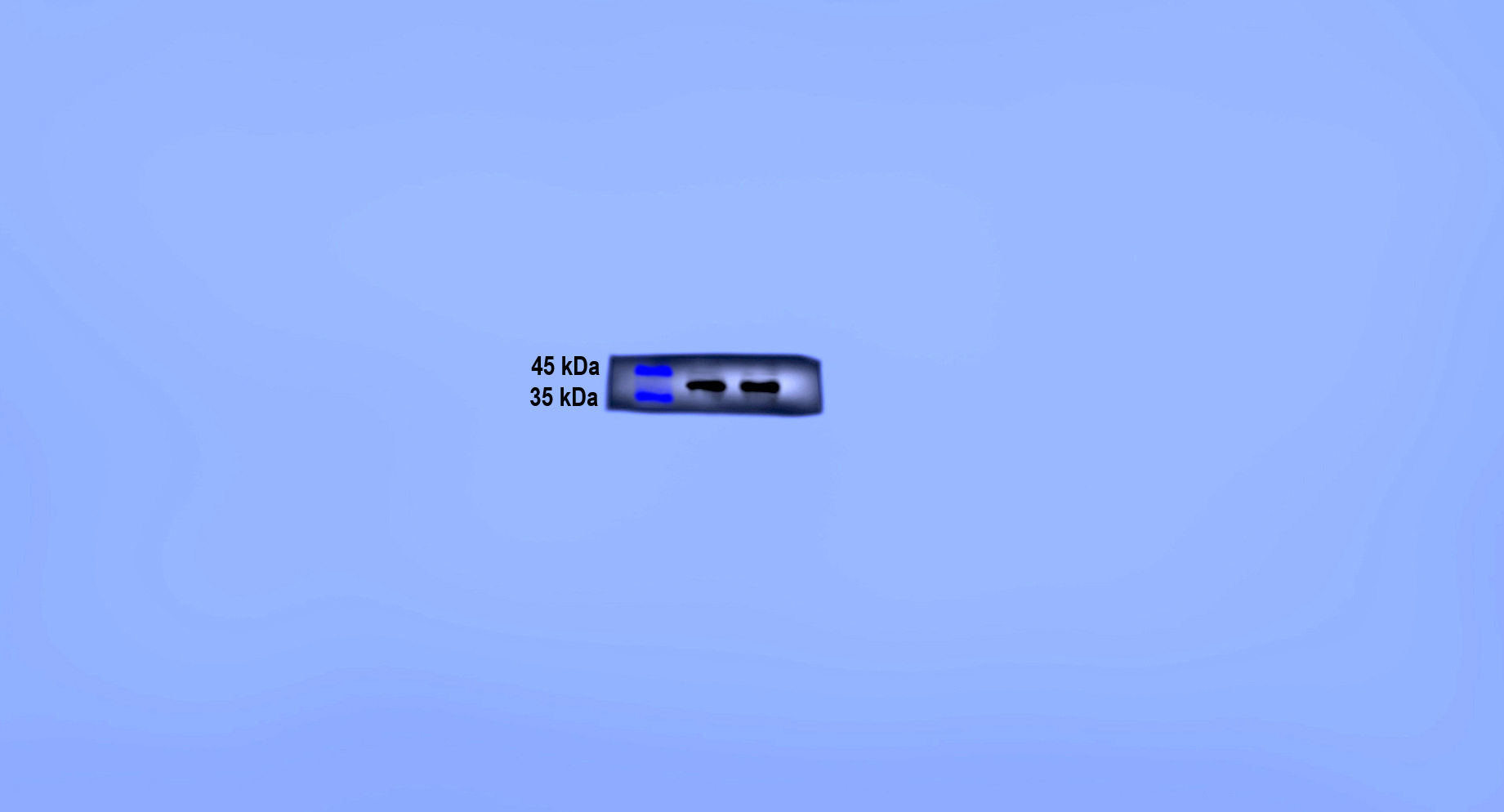

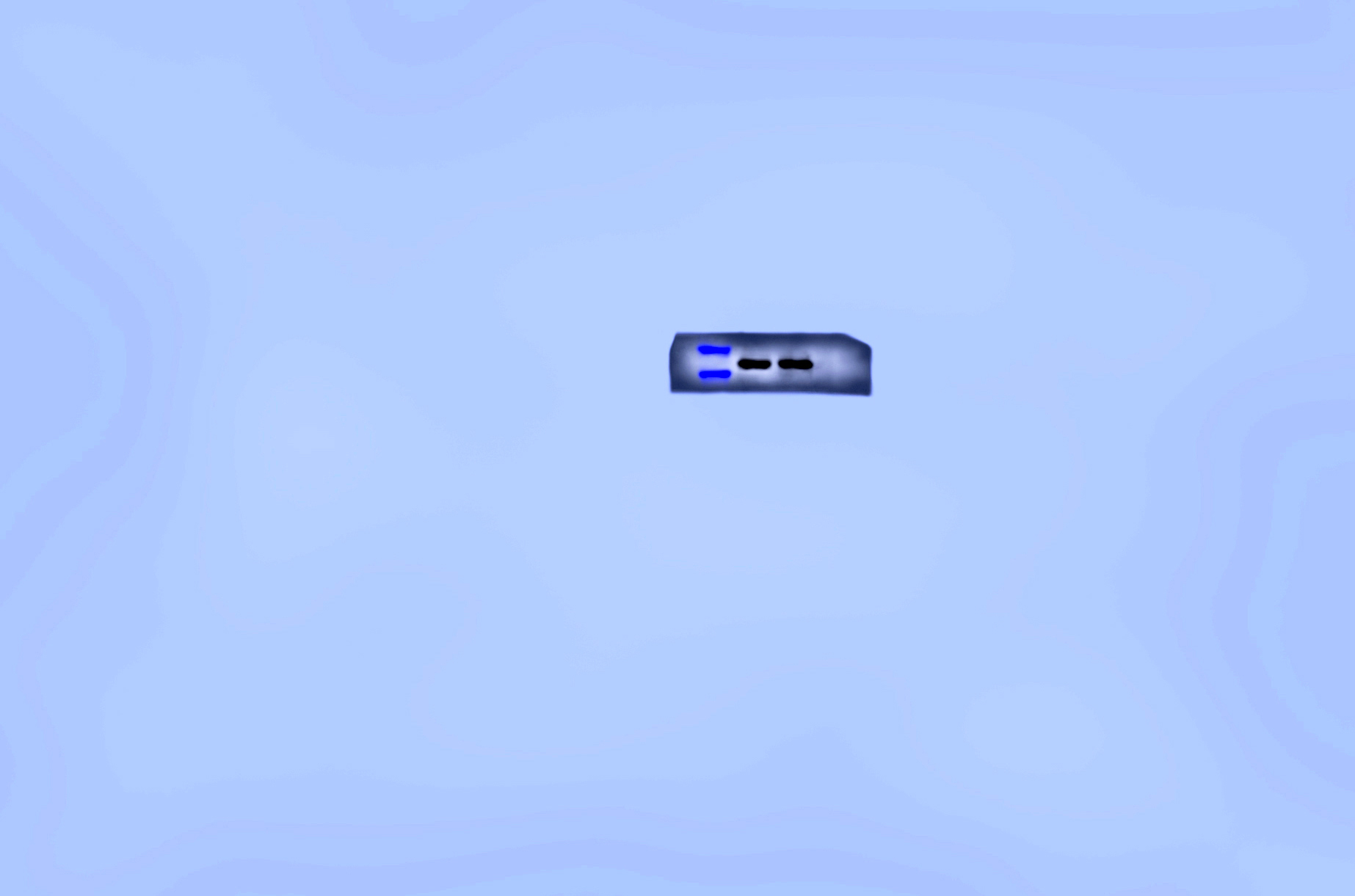

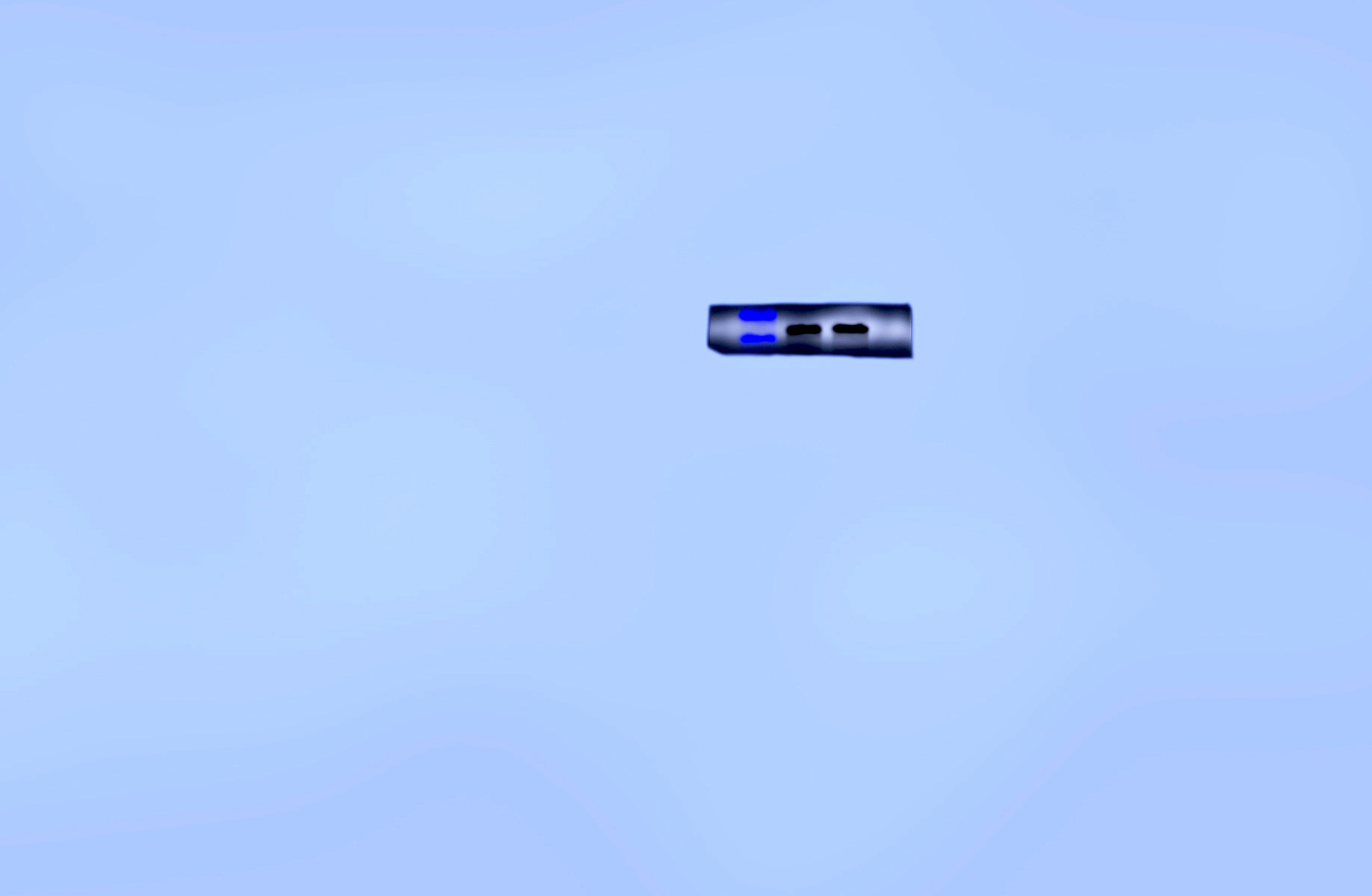


**Figure 1B**

LC3II

Repeat 1 Repeat 2 Repeat 3


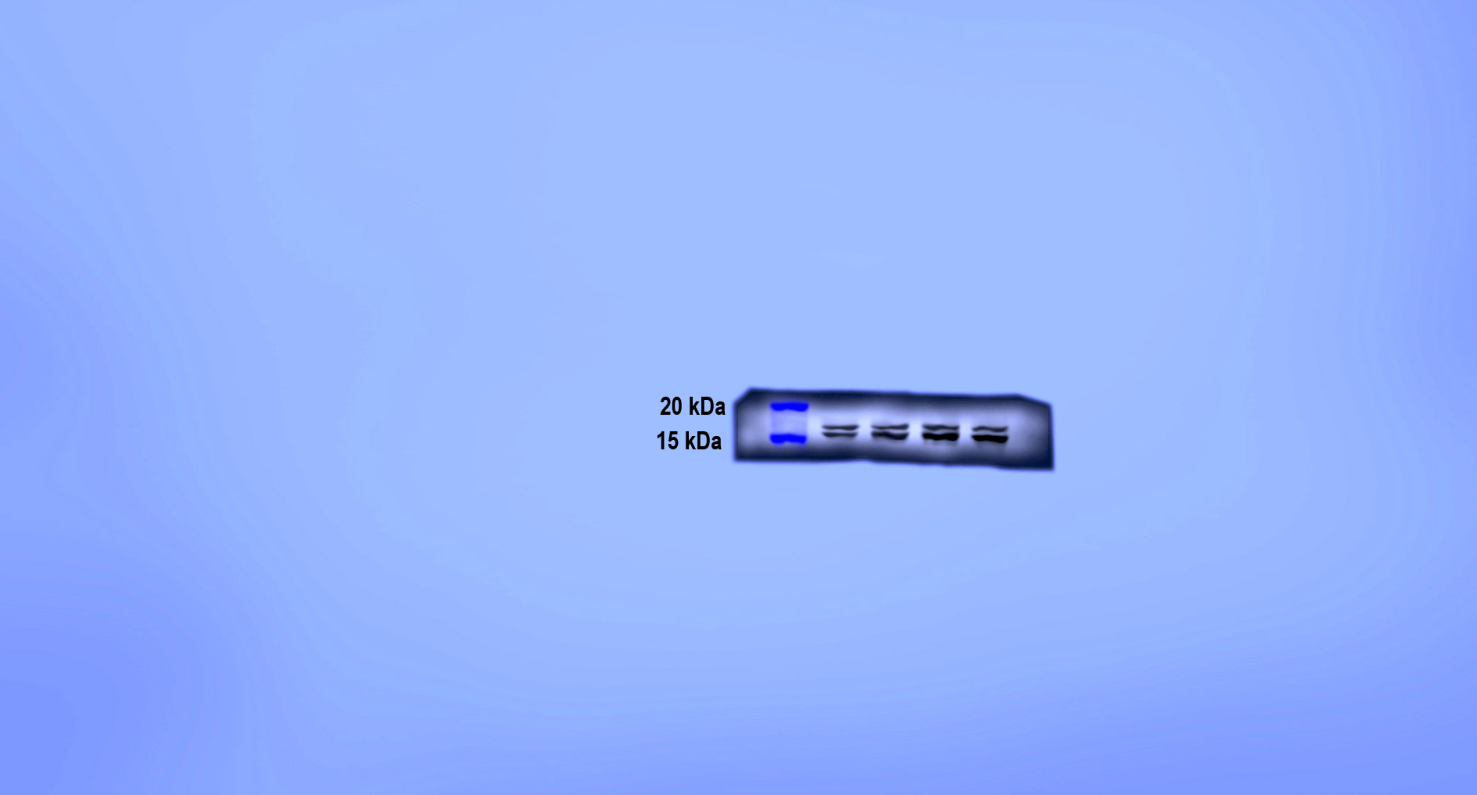

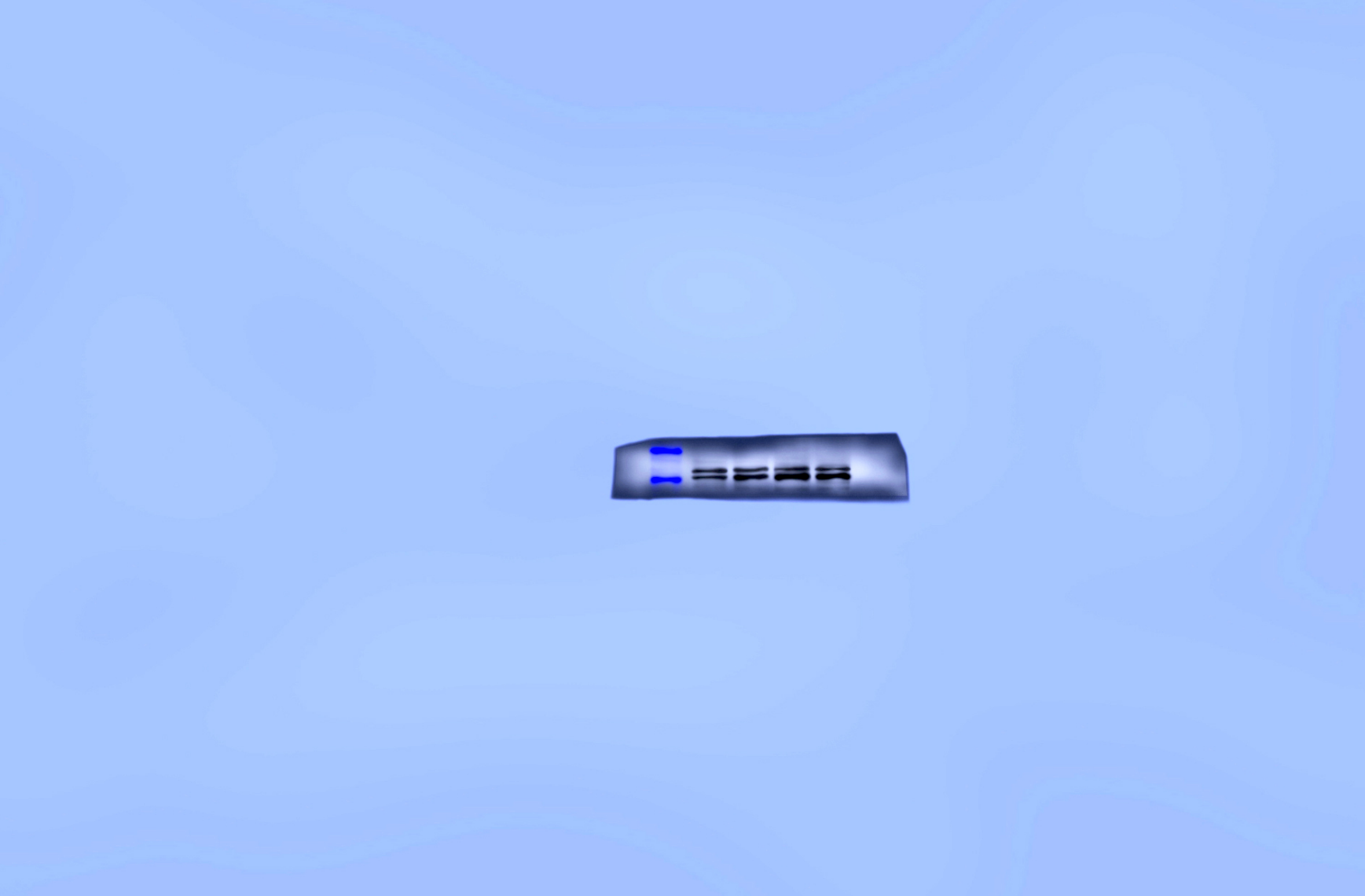

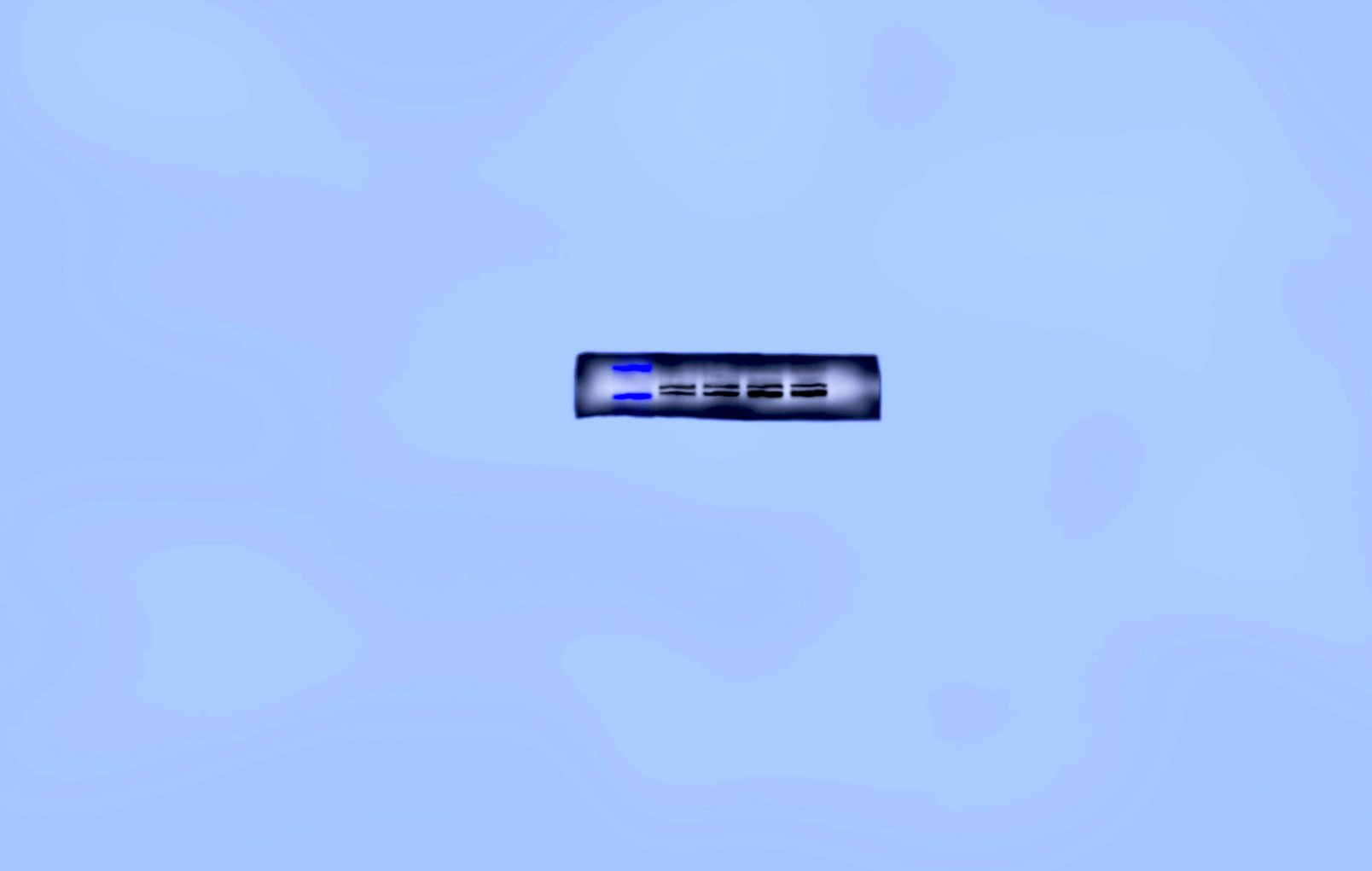


GAPDH

Repeat 1 Repeat 2 Repeat 3


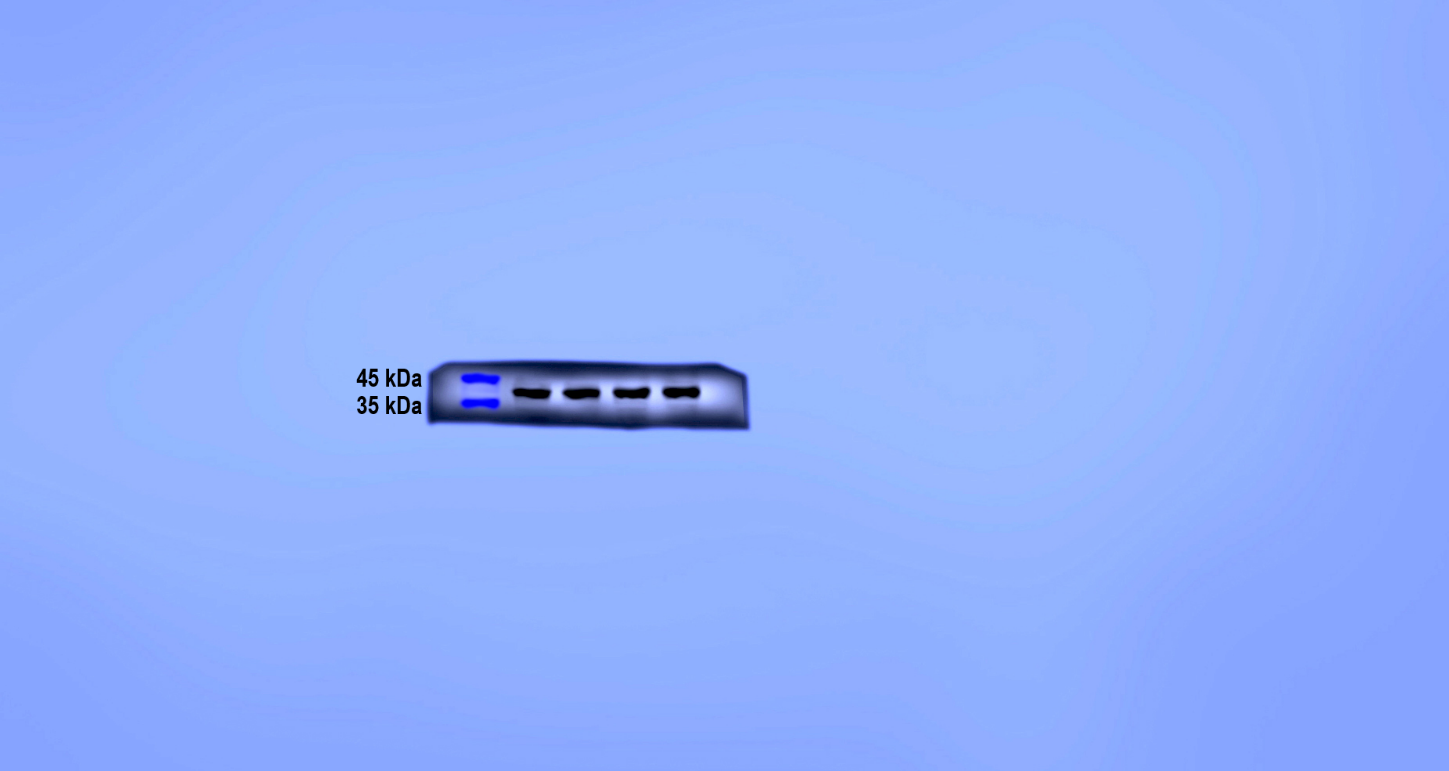

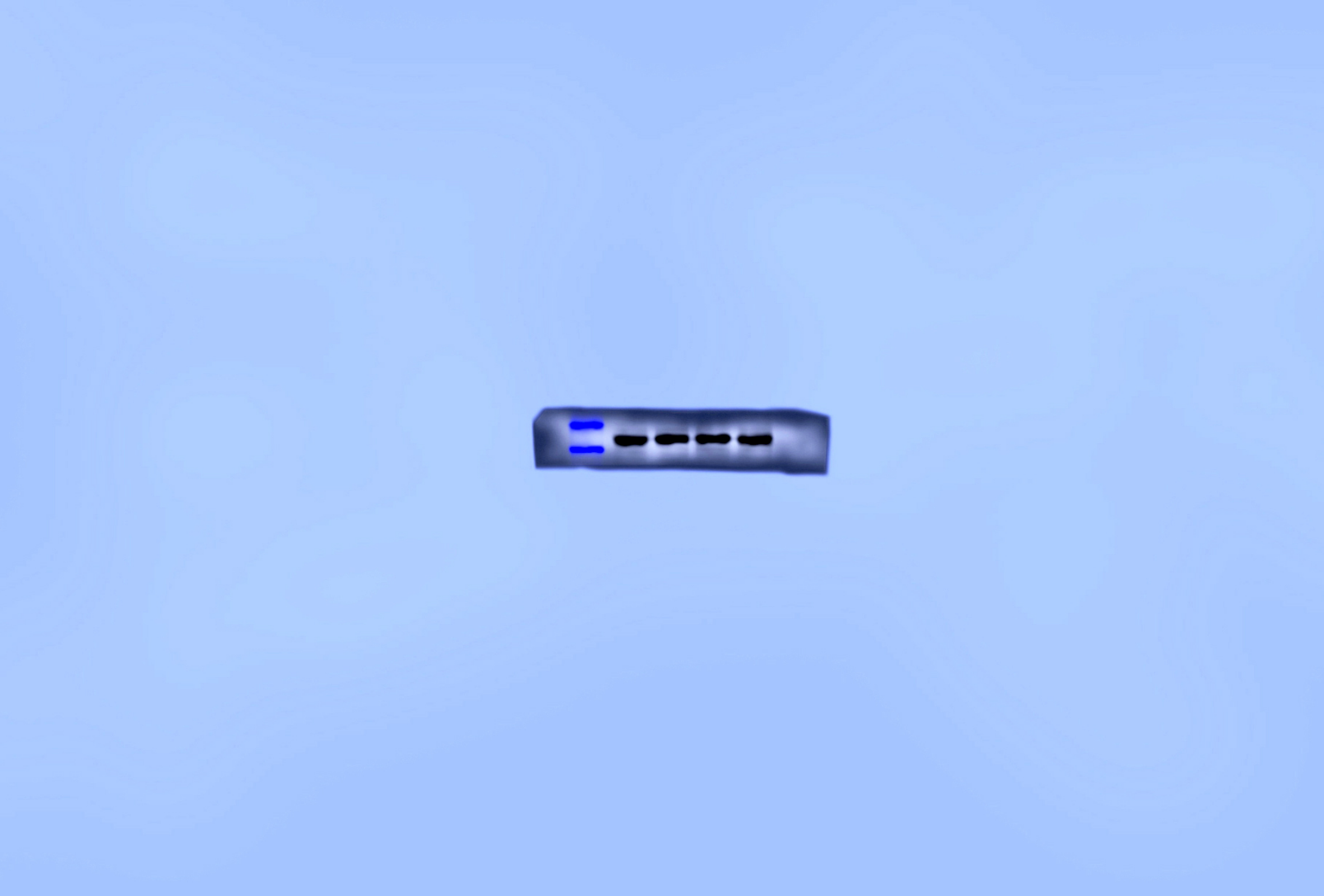

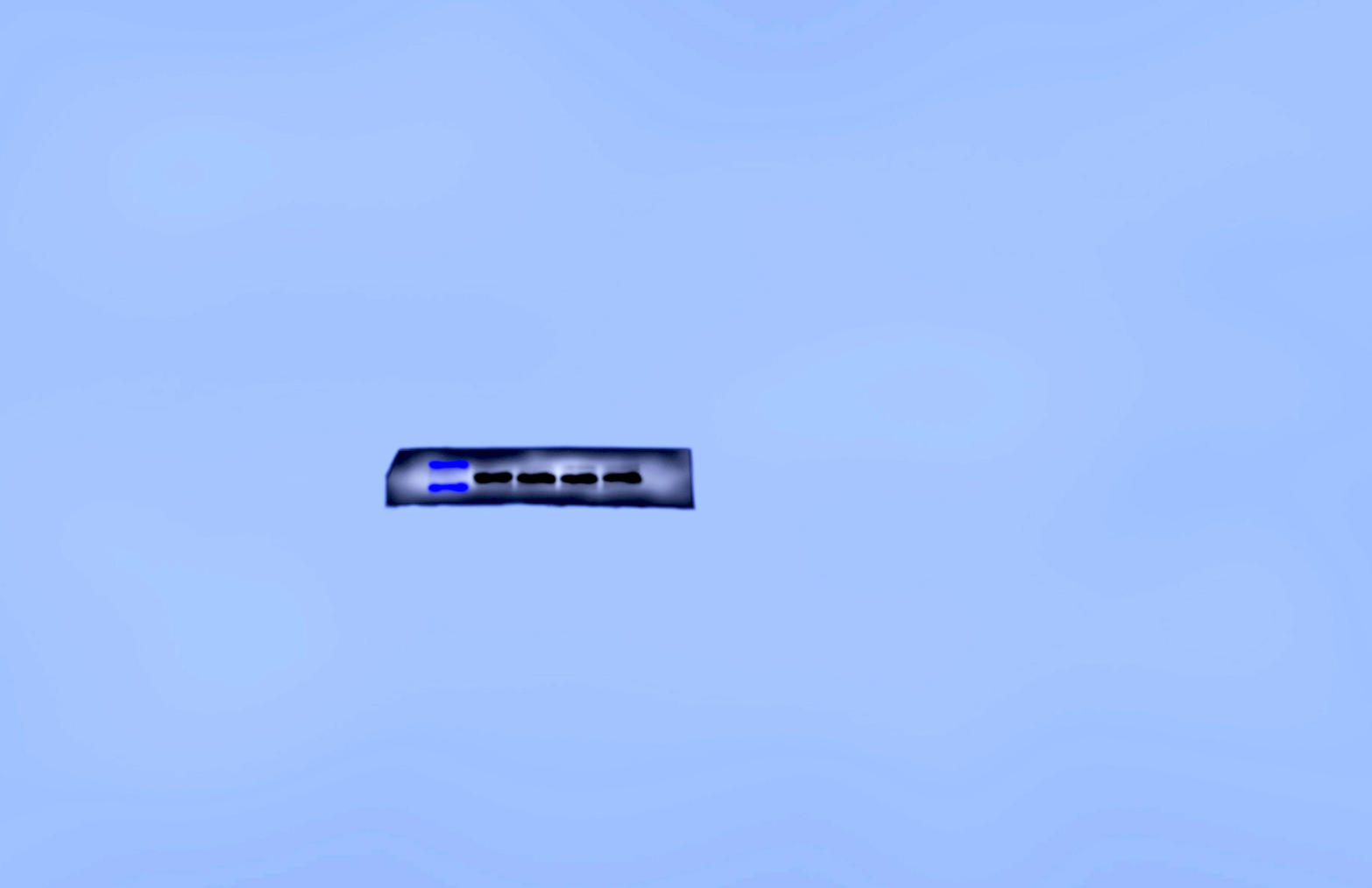


**Figure 3A**

LC3II

Repeat 1 Repeat 2 Repeat 3


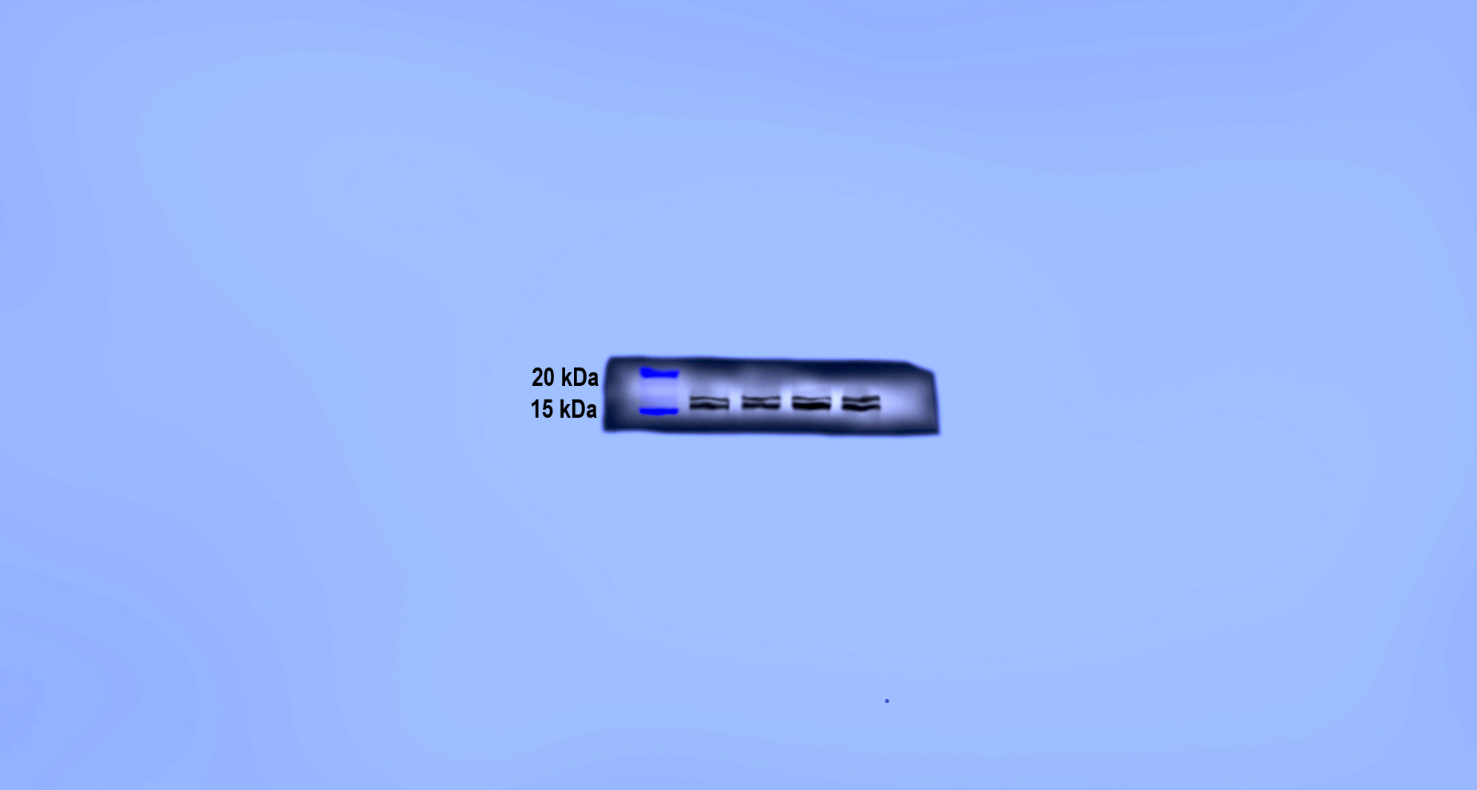

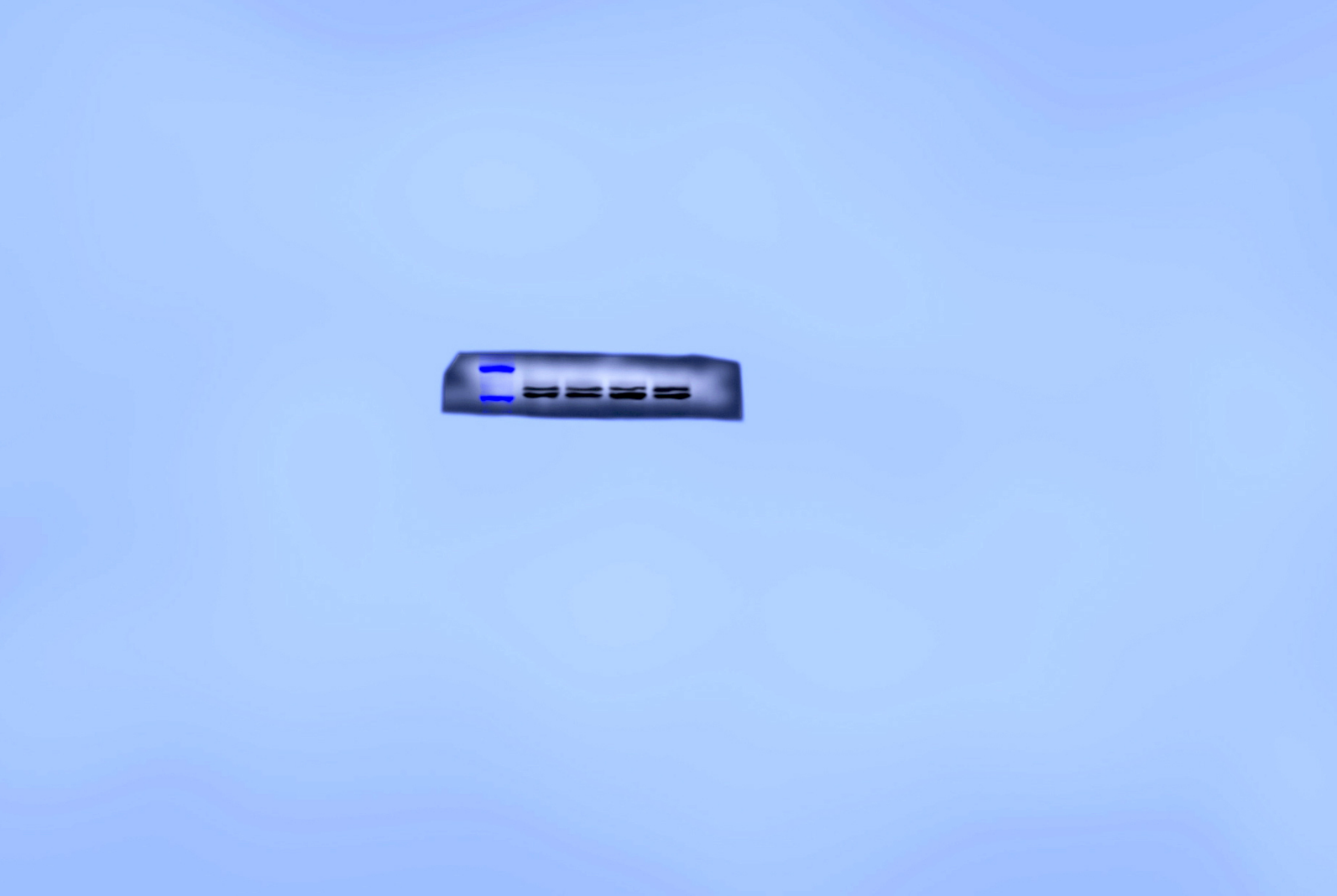

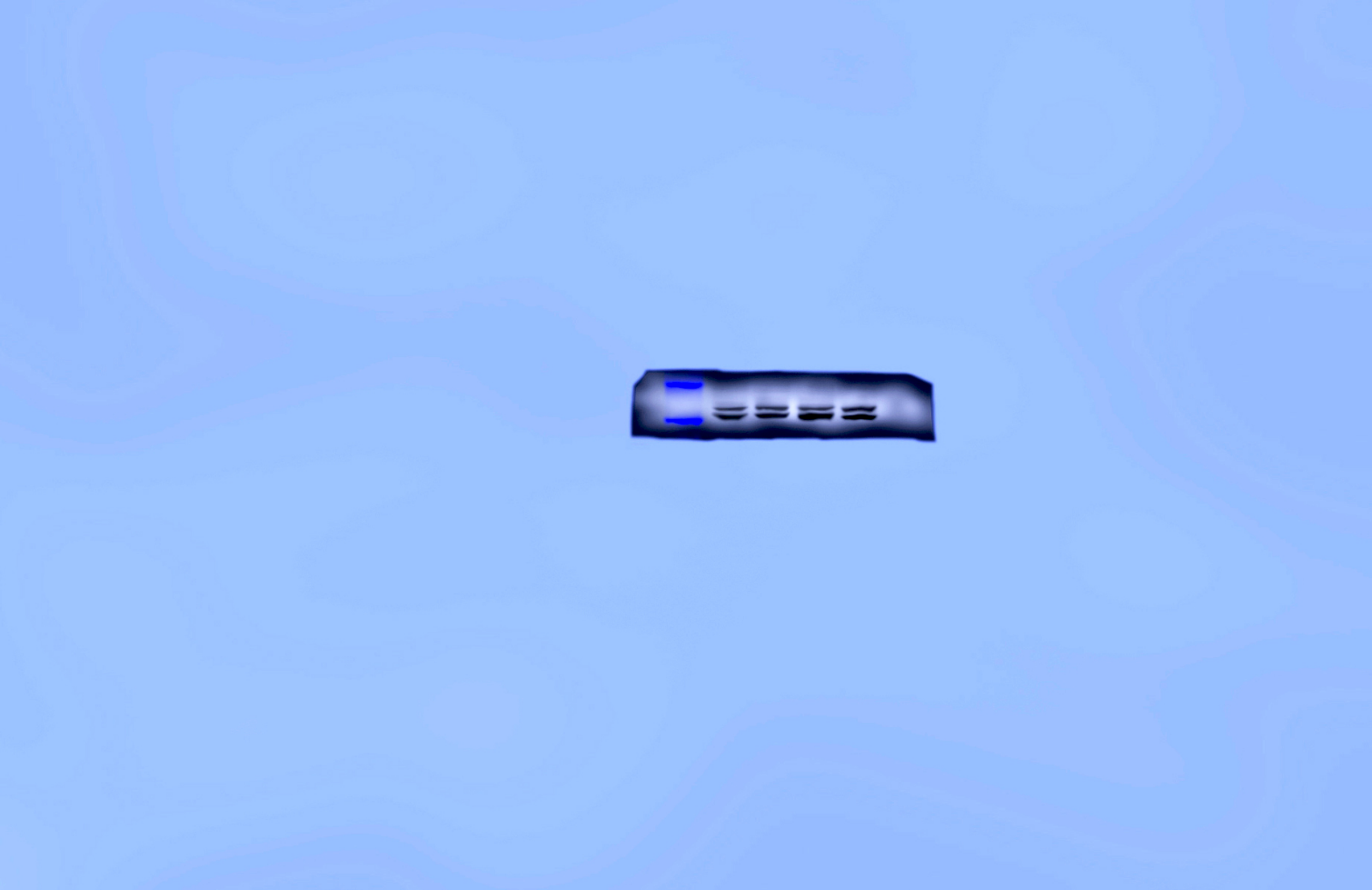


P62

Repeat 1 Repeat 2 Repeat 3


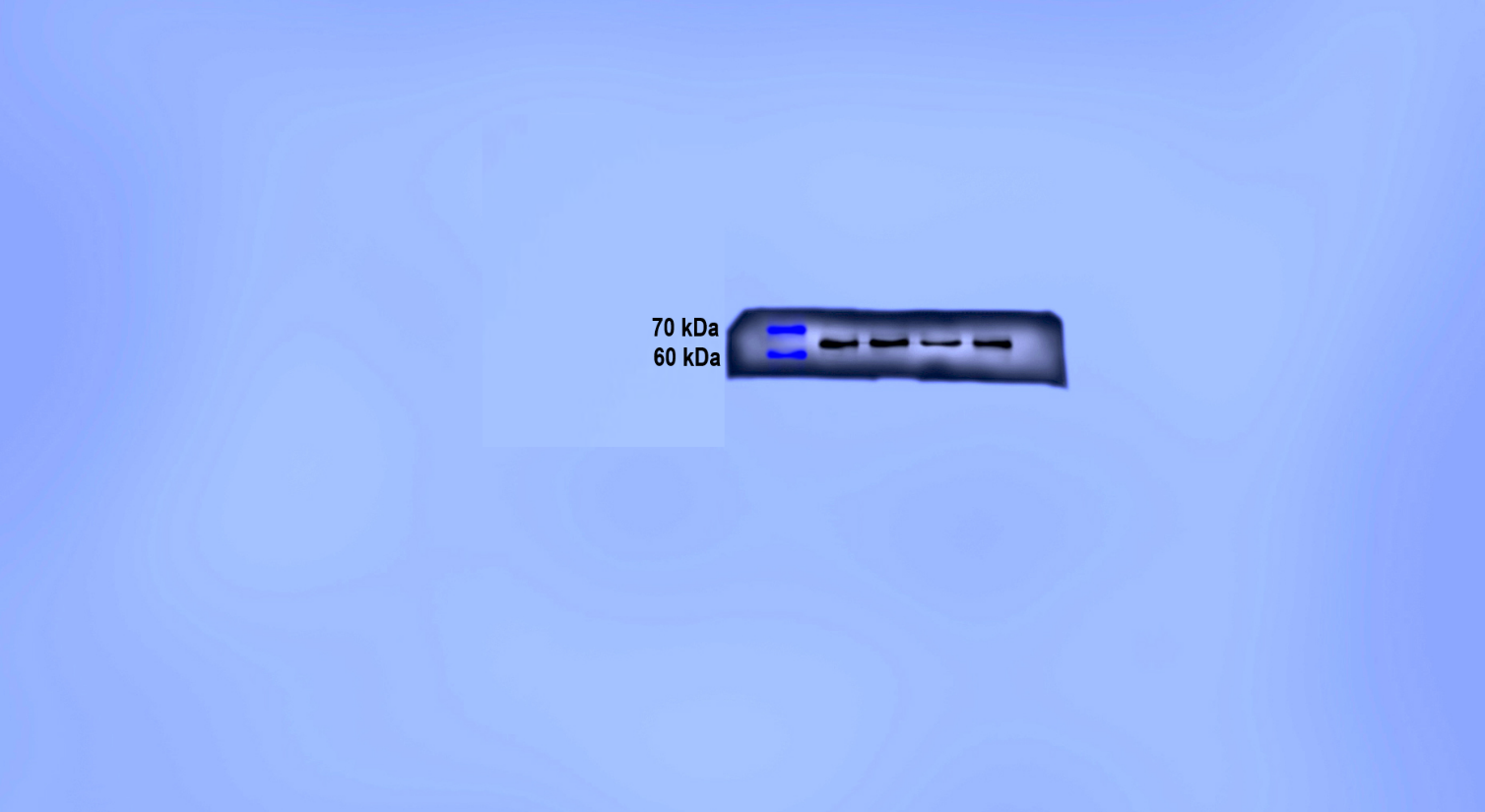

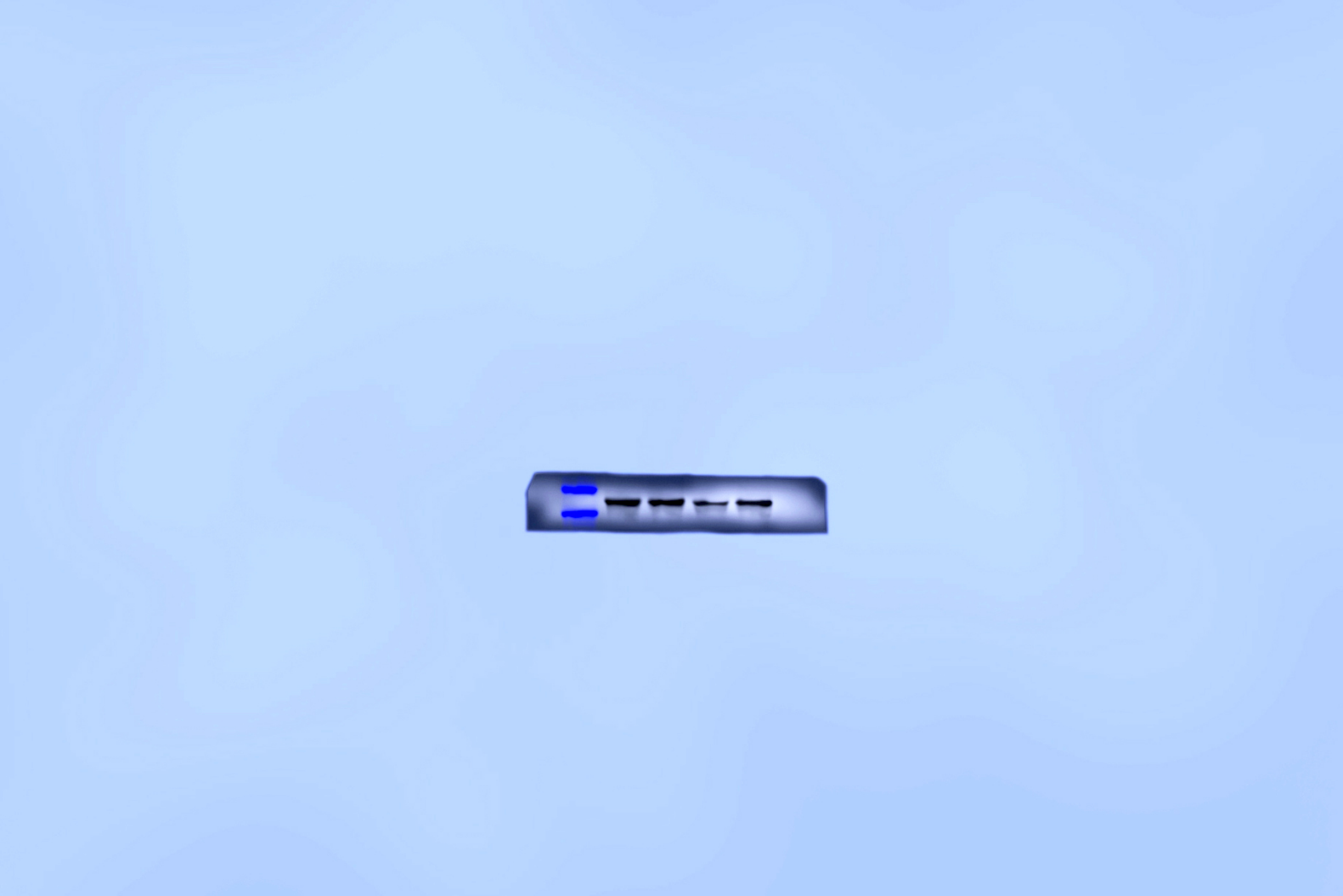

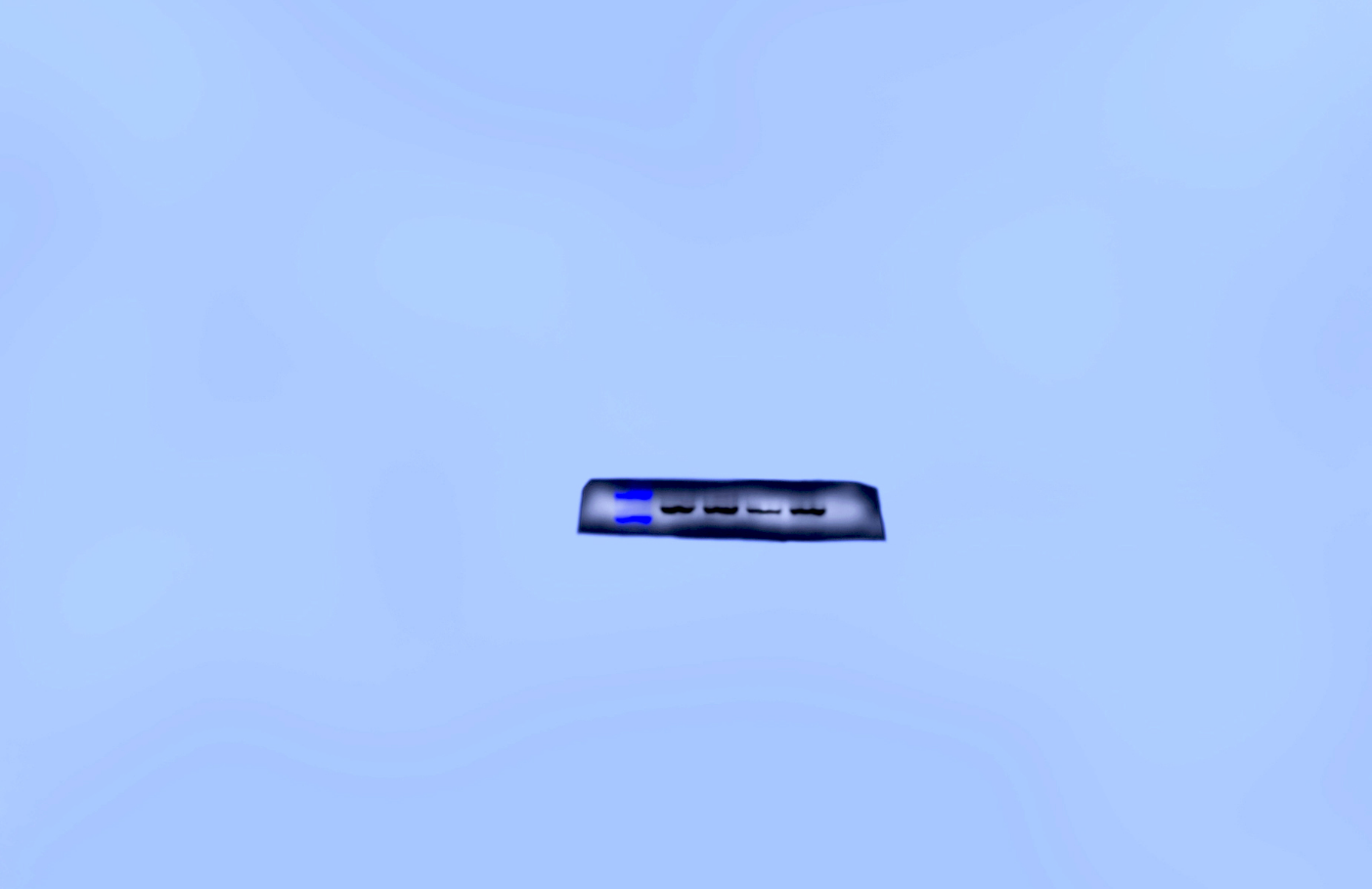


GAPDH

Repeat 1 Repeat 2 Repeat 3


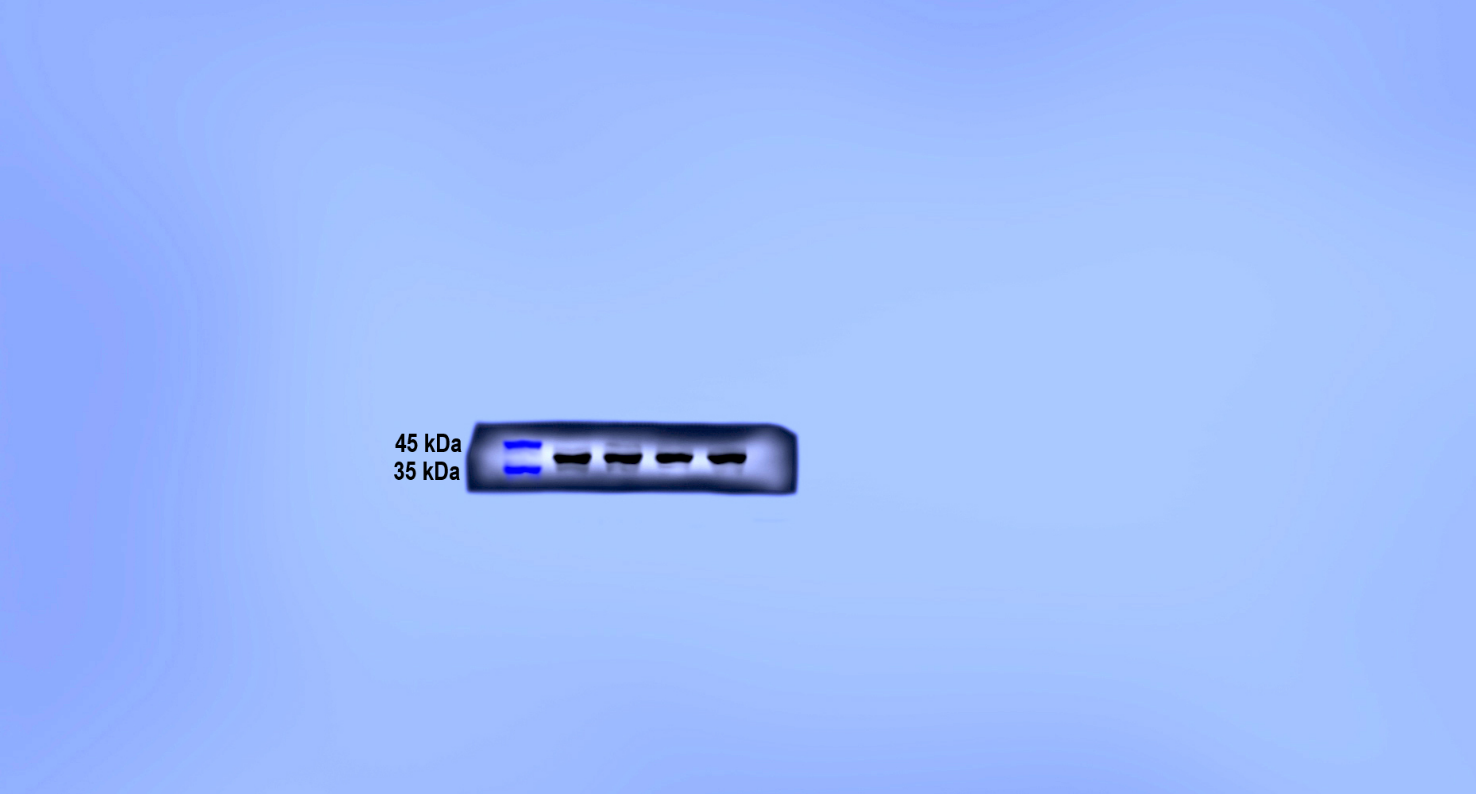

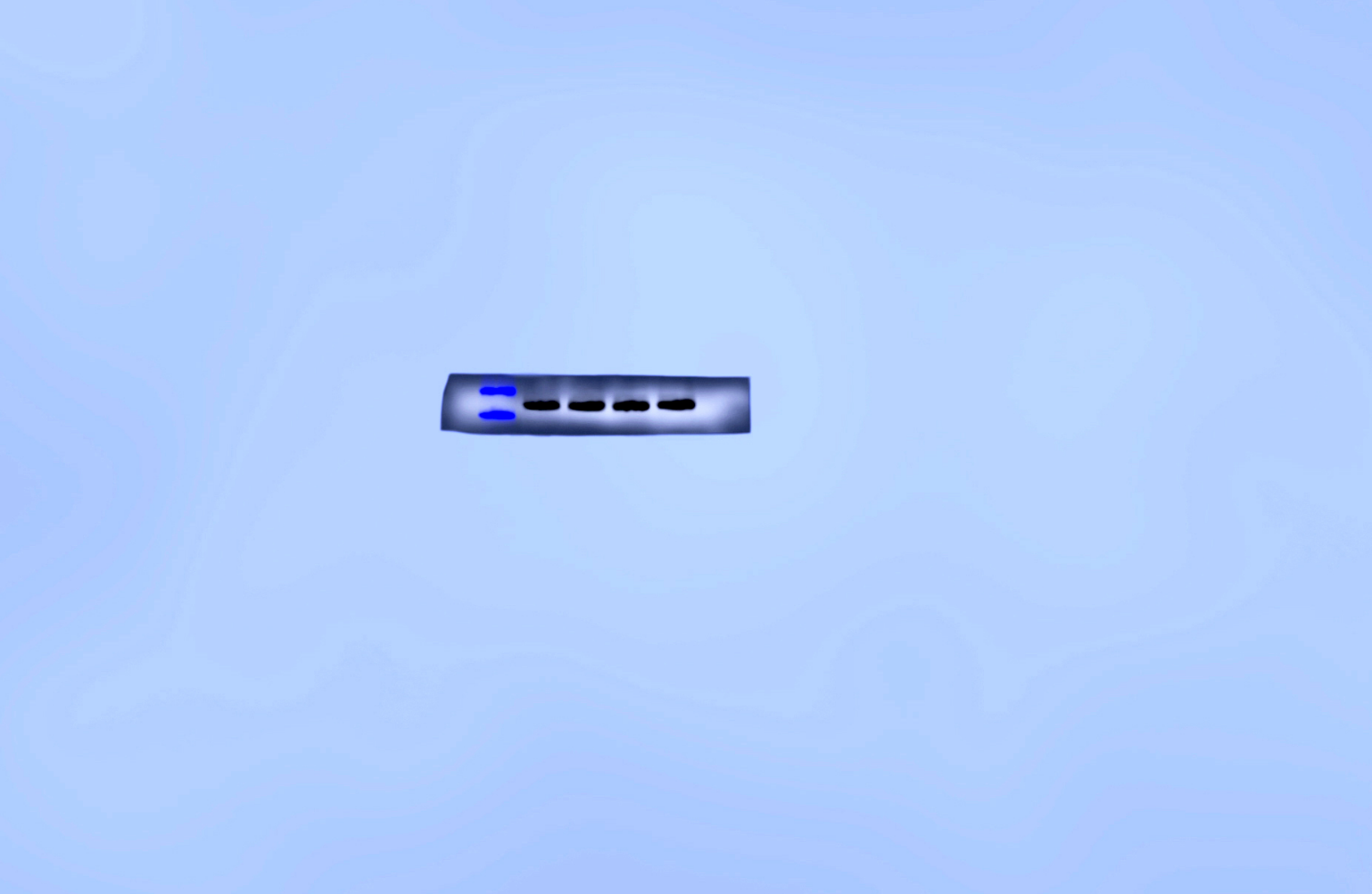

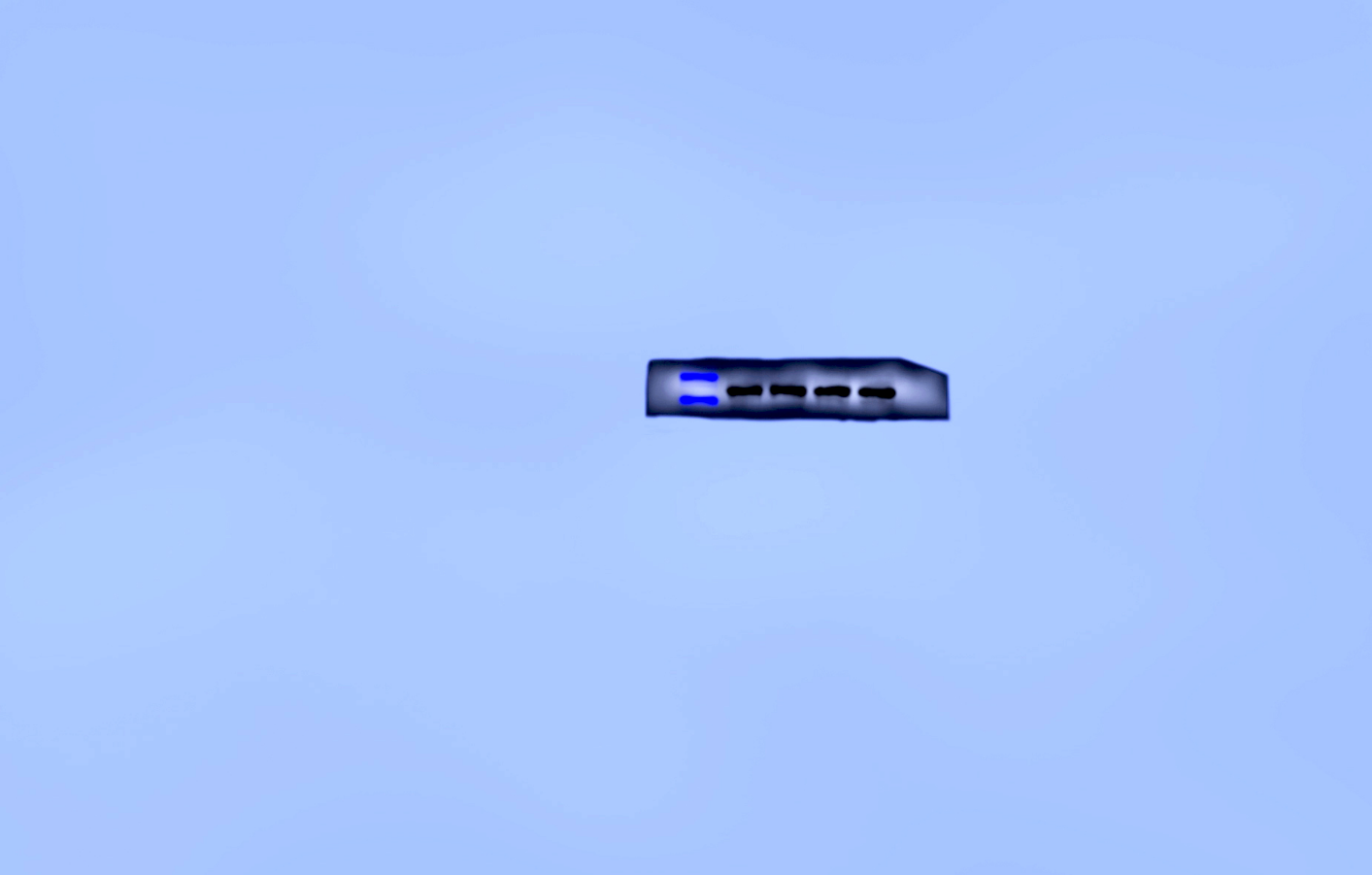

Supplement: Supplementary file 1 — Supplementary Material 1 [file 12872_2023_3496_MOESM1_ESM.docx]
